# *Leishmania donovani* Secretory Mevalonate Kinase Regulates Host Immune Response and Facilitates Phagocytosis

**DOI:** 10.3389/fcimb.2021.641985

**Published:** 2021-04-26

**Authors:** Tanvir Bamra, Taj Shafi, Sushmita Das, Manjay Kumar, Manas Ranjan Dikhit, Ajay Kumar, Ashish Kumar, Kumar Abhishek, Krishna Pandey, Abhik Sen, Pradeep Das

**Affiliations:** ^1^ Department of Molecular Biology, ICMR–Rajendra Memorial Research Institute of Medical Sciences, Patna, India; ^2^ Department of Microbiology, AIIMS, Patna, India

**Keywords:** *Leishmania*, mevalonate kinase, immune response, phosphorylation, infection, secretion, invasion, phagocytosis

## Abstract

*Leishmania* secretes over 151 proteins during *in vitro* cultivation. Cellular functions of one such novel protein: mevalonate kinase is discussed here; signifying its importance in *Leishmania* infection.

Visceral Leishmaniasis is a persistent infection, caused by *Leishmania donovani* in Indian subcontinent. This persistence is partly due to phagocytosis and evasion of host immune response. The underlying mechanism involves secretory proteins of *Leishmania* parasite; however, related studies are meagre. We have identified a novel secretory *Leishmania donovani* glycoprotein, Mevalonate kinase (MVK), and shown its importance in parasite internalization and immuno-modulation. In our studies, MVK was found to be secreted maximum after 1 h temperature stress at 37°C. Its secretion was increased by 6.5-fold in phagolysosome-like condition (pH ~5.5, 37°C) than at pH ~7.4 and 25°C. Treatment with MVK modulated host immune system by inducing interleukin-10 and interleukin-4 secretion, suppressing host’s ability to kill the parasite. Peripheral blood mononuclear cell (PBMC)-derived macrophages infected with mevalonate kinase-overexpressing parasites showed an increase in intracellular parasite burden in comparison to infection with vector control parasites. Mechanism behind the increase in phagocytosis and immunosuppression was found to be phosphorylation of mitogen-activated protein (MAP) kinase pathway protein, Extracellular signal-regulated kinases-1/2, and actin scaffold protein, cortactin. Thus, we conclude that *Leishmania donovani* Mevalonate kinase aids in parasite engulfment and subvert the immune system by interfering with signal transduction pathways in host cells, which causes suppression of the protective response and facilitates their persistence in the host. Our work elucidates the involvement of *Leishmania* in the process of phagocytosis which is thought to be dependent largely on macrophages and contributes towards better understanding of host pathogen interactions.

## Introduction

The Leishmaniases are a set of neglected tropical diseases caused by 20 species of *Leishmania* parasites and is transmitted to humans by infected female sandfly bite. Among different types of the disease caused by *Leishmania*, Visceral Leishmaniasis (VL) is the most severe one which is often fatal if not treated in time. In India, VL is caused by *Leishmania donovani* species. Progresses in controlling the Leishmaniases will require better understanding of pathogenesis to recognize novel drug targets or vaccine candidates.

*Leishmania* exists in two forms; the extracellular, flagellated, motile form is promastigote, that resides in the alimentary canal of the sandfly. Blood feeding activity of the vector results in the transmission of the parasite to the human where it is phagocytosed and transformed into intracellular, non-flagellated, non-motile amastigote form. *Leishmania* is an enormously successful organism considering its two structural variants and ability to exist in the harsh host environment. Some of these survival mechanisms may be attributed to a large repertoire of proteins secreted by the parasite. *L. donovani* releases a total of 151 proteins in abundance to the extracellular media (Silverman et al., 2008). These are not a set of unrelated proteins, rather, these are functionally related group of proteins (Geiger et al., 2010). The exoproteome is known to assist the entry of parasite into host cells which is a prerequisite for infection (Nandan et al., 2002; Choudhury et al., 2010a; Zylbersztejn et al., 2015). In addition, *Leishmania* exosome treatment induces immune suppression of macrophage prior to infection and creates an environment to support early infection (Silverman et al., 2010). The secretory proteins and surface molecules on parasites form an interface between the parasite and host. Though, the major cell surface molecules of parasites are well characterized (Connell et al., 1993; McConville et al., 1993; Winter et al., 1994), very less information is available regarding secretory proteins/antigens for their role in host infection.

Mevalonate kinase, one such secreted protein has been reported with different organisms. However, Mevalonate kinase in *Leishmania donovani* is not known. Mevalonate pathway, present in most of the eukaryotic cells, is essential for various cellular functions, such as, cell cycle regulation, control of cell growth and size, autophagy, and protein glycosylation (Fu et al., 2002; Miettinen and Björklund, 2016). The mevalonate pathway also provides precursors for cholesterol biosynthesis. Mevalonate kinase (MVK) is an important enzyme of this pathway catalyzing Mg^2+^-ATP dependent phosphorylation of mevalonic acid to mevalonate-5-phosphate. This step is regulated by feedback inhibition (Dorsey and Porter, 1968; Henneman et al., 2011). *L. major* MVK crystal structure was elucidated and its ATP binding site was found to be structurally distinct (Sgraja et al., 2007). In *Trypanosoma cruzi*, MVK is secreted outside the cell and it modulates the host cell signaling (Ferreira et al., 2016). In *L. major* and *T. cruzi*, dimeric MVK has a high enzymatic activity (Sgraja et al., 2007; Ferreira et al., 2016). However, the mevalonate pathway in *L. donovani* has not been studied till date.

Here, we have demonstrated that MVK protein is present in *L. donovani* and is secreted. It was observed that it regulates host immune response and induce parasite entry through phosphorylation of ERK-1/2, p-38, and cortactin. Altogether, our work sheds light on the involvement of *Leishmania* in the process of phagocytosis and contributes towards better understanding of host pathogen interactions.

## Material and Methods

### Ethics

All experiments were assessed and approved by the Institutional Animal Ethical Committee (AH/RMRIMS/IAEC/09/33-37), Indian Council of Medical Research, Rajendra Memorial Research Institute of Medical Sciences, Patna, and are managed by CPCSEA (The Committee for the Purpose of Control and Supervision of Experiments on Animals), Government of India, New Delhi. ICMR-RMRIMS (Indian Council of Medical Research- Rajendra Memorial Research Institute of Medical Sciences) follows “The Guide for the Care and Use of Laboratory Animals,” were followed as per 8th edition by the Institute for Laboratory Animal Research. For the use of human sample too, approval from ethical committee was taken (RMRI/EC/02/20).

### Parasites

Ag83 strain of *L. donovani* originally obtained from an Indian VL patient was maintained routinely in mice as earlier described (Das et al., 2012). Promastigotes were maintained in Gibco™ Medium-199 (ThermoFisher Scientific; #10063372) containing 10% (v/v) heat-inactivated Gibco™ fetal bovine serum (FBS; ThermoFisher Scientific; #10082139), 25 mM 4-(2-hydroxyethyl)-1-piperazineethanesulfonic acid (HEPES) (Calbiochem), 4 mM sodium bicarbonate (NaHCO_3_) (Sigma-Aldrich), 100 µg/ml streptomycin (Sigma-Aldrich), and 100 units/ml penicillin G-sodium (Sigma-Aldrich) at 24°C as earlier described (Kumar et al., 2018).

### Generation of Axenic Amastigotes

Axenic amastigotes were generated as described previously (Kaul et al., 2000) with minor modifications. Promastigote culture grown in Medium199 supplemented with 20% FBS was incubated at 37°C. After 24 h, cells were maintained in Medium199 (20% fetal bovine serum) with pH 5.5 under same temperature conditions for 5 days. Obtained axenic amastigote culture were used for the experiments.

### Signal Peptide and Transmembrane Protein Prediction

Protein sequence of LdMVK was obtained from sequenced MVK gene in FASTA format and uploaded in SecretomeP 2.0 server to study the possibility of its secretion by non-classical i.e. non-signal peptide triggered protein secretory route. Due to the limited organism group in list, mammalian was selected as the organism group and the sequence was submitted.

### Polymerase Chain Reaction Amplification

Full length LdMVK gene coding region was amplified from the genomic deoxy-ribonucleic acid (DNA) of Ag83 strain using following primer pair: Forward- 5’TTTTGGATCCATGCCAAAGCCCGTCAAG3’ and Reverse- 5’TTTTAAGCTTCAGGTTTGACGCGGTGG3’. Polymerase Chain Reaction **(**PCR) conditions were: 60 s at 94°C, 45 s at 56°C, 60 s at 72°C (35 cycles), and 10 min at 72°C for final extension. Full length MVK gene sequence was obtained through di-deoxy sequencing of MVK-PCR product.

### Soluble *Leishmania* Antigen Preparation

To prepare Soluble *Leishmania* antigen (SLA), promastigotes harvested from 3 to 4 days of culture was washed two times in PBS and resuspended in PBS containing protease inhibitors cocktail (Sigma). Ultra-sonication was performed followed by centrifugation (20,000g for 20 min) and protein quantification of the supernatant was done by Bicinchonic acid method and SLA was stored at −20°C for further use.

### Supernatant Processing and Determination of Mevalonate Kinase Activity

*Leishmania* was inoculated at a concentration of 1 × 10^6^ cells/ml. Promastigotes and amastigotes of 4^th^ day culture were washed three times with 1X PBS and incubated in pre-warmed (37°C) Medium199 (without FBS) containing Halt protease inhibitor cocktail (Thermo Scientific) at a concentration of 1 × 10^8^ cells/ml for required time. The viability of parasites was assessed by trypan blue dye exclusion test to ensure over 98% viability. The culture was centrifuged (3,000 g, 10 min, 4°C) and the obtained supernatant was passed through 0.45 μm pore size syringe filters (Millipore) to remove any remaining parasites. It was then concentrated at 4°C to 100 μl volume by 10 kDa MWCO (Molecular weight cut off) centricon (Millipore) according to the manufacturer’s protocol. Distilled water was added to the concentrated sample to reduce the salt and phenol red content and sample was again concentrated to required volume. Wherein required, the obtained supernatant consisting of secretory proteins was quantified by bicinchonic acid assay and then stored at −20°C until use. Once obtained, the supernatant was kept on ice at all times.

For time kinetics experiments and heat and pH based relative studies, normalization of the detected MVK amount could not be done with other secretory protein since there are no known proteins that are constitutively secreted from parasites in a stable manner. Therefore, the parasites were carefully counted for each sample before supernatant preparation and soluble *Leishmania* antigen was prepared by already described method. For normalizing blots, both cell lysate and supernatants for the same population was run and the protein of interest was normalized against cell number control (β-actin).

To check the presence of mevalonate kinase activity in culture supernatant, *L. donovani* promastigotes were incubated in serum-free Medium199 for 6 h at 37°C. Culture supernatant was collected, concentrated, and mevalonate kinase assay was performed. Reaction mixture was prepared consisting of 100 mM glycine, 25 mM sodium chloride (NaCl) pH 9.0, 4 U lactic dehydrogenase, 4 U pyruvate kinase, 1 mM phosphoenolpyruvate, 5 mM adenosine triphosphate (ATP), 5 mM Magnesium chloride (MgCl_2_), 4 mM mevalonate, and 30 μM β-nicotinamide adenine dinucleotide hydrogen (β-NADH). It was incubated at 25°C for 10 min and assay was initiated by concentrated supernatant addition (0.6 µg/µl; 260/280: 0.63). MVK activity was examined by coupling ADP release with oxidation of NADH by pyruvate kinase and lactate dehydrogenase, which was measured at 340 nm for 600 s (time scan) using Shimadzu UV-visible spectrophotometer. For blank assays, Medium199 processed using centricon was used. The reactions were performed in triplicates and culture supernatants from different batches were used for each assay.

### Cloning, Expression, Purification, and Polyclonal Antisera Generation Against LdMVK

The PCR product was ligated to pET-28a expression vector (Novagen). pET28a is a bacterial expression vector that expresses protein in fusion with amino terminal and carboxy terminal His_6_ tag. Recombinant vector and PCR fragment was sequenced by dideoxynucleotide chain termination method for confirmation of cloning. *E. coli* pET-28a-LdMVK vector were transformed in BL-21(DE3) cells, grown at 37°C, 150 rpm and 1 mM IPTG induction was performed for 4 h. Cells were resuspended in buffer containing 50 mM Tris-HCl, 300 mM sodium chloride (NaCl), 1X protease inhibitor cocktail (Roche), and 0.1 mg/ml of lysozyme (Sigma) and incubated in ice bath for 30 min. Sonication disrupted cells and debris was removed by centrifugation (20,000 g, 15 min). Expression of protein was evaluated by western blot using anti-his antibody (1:1,000 dilution; ThermoFisher Scientific; #MA1-135).

For affinity chromatography purification, cell lysate was passed through Nickel-nitrilotriacetic acid (Ni-NTA) resin column (Qiagen). Protein was eluted from the nickel column with an increasing buffer gradient consisting of 150 mM Tris pH-7.8, 300 mM NaCl, and 250 mM imidazole. Anti-LdMVK antibodies were generated by one subcutaneous immunizations with pure rLdMVK along with complete Freund’s adjuvant (Sigma-Aldrich, St. Louis, CA, USA), following three subcutaneous immunizations along with incomplete Freund’s adjuvant (Sigma-Aldrich, St. Louis, CA, USA) in rabbit at 14-day intervals. Serum was isolated 10 days after last immunization and western blot of rLdMVK and Ag83 soluble *Leishmania* antigen were performed to verify antibody specificity.

### Ouchterlony Test

Gel slides were prepared using 1.2% agarose in assay buffer and poured 4 ml/slide. Wells were bore on gel. Then 10 µl rLdMVK protein was placed on the well at the center and 10 µl each of post immune sera, distilled water, and pre-immune sera was placed the other wells. The slide was incubated overnight in a moist chamber at 37°C and the next day it was observed.

### Western Blotting

To confirm the expression of recombinant MVK (containing his-tag in N and C-terminal), expressed bacterial cell lysate was separated on 12% SDS-PAGE gel and probed with anti-his antibody at 1:1,000 dilution for 1h and then with anti-rabbit antibody (1:5,000 dilution; Jackson ImmunoResearch laboratories; AB_2307391) for 45 mins. For secretion experiments, culture supernatant of *L. donovani* was concentrated and run in SDS-PAGE. Secretory proteins were transferred onto PVDF membrane and processed for western blots analysis with anti-MVK antibody raised in rabbit at 1:1,000 dilutions for 1 h and then with anti-rabbit antibody (1:5,000 dilution; Jackson ImmunoResearch laboratories; AB_2307391) at 1:5,000 dilutions for 45 mins. Antibody antigen complexes were detected by enhanced chemiluminescence (GE Healthcare; #RPN2209) and bands were visualized using Image J software. Band densitometry was performed using ImageJ software. To validate that the supernatant containing secretory proteins was collected from equal number of cells, cell lysate was prepared for each condition and probed with anti-actin antibody which was kindly provided by Dr Anuradha Dube.

### Characterization of MVK

#### Enzymatic Activity of r-LdMVK

Specific activity of purified r-LdMVK was tested. Reaction mixture consisting of 100 mM glycine, 25 mM sodium chloride (NaCl) pH 9.0, 4 U lactic dehydrogenase, 4 U pyruvate kinase, 1 mM phosphoenolpyruvate, 5 mM adenosine triphosphate (ATP), 5 mM Magnesium chloride (MgCl_2_), 4 mM mevalonate, and 30 μM β-nicotinamide adenine dinucleotide hydrogen (β-NADH) were incubated at 25°C for 10 min. To start the reaction, r-LdMVK was added, cuvette with lid was inverted for mixing, and time scan was performed immediately at 340 nm for 600 s to monitor NADH oxidation. Mevalonate kinase activity converts ATP to ADP which is used by pyruvate kinase to form pyruvate. Pyruvate, in the presence of pyruvate kinase, is converted to lactate, oxidizing NADH. Thus, NADH oxidation is linked to mevalonate kinase activity. Blank assays were performed in r-LdMVK absence. Enzyme activity of one unit corresponds to production of 1 mol NADH/min. Specific activity was expressed as µmole product formed/min/mg protein. The reactions were done in triplicates and purified proteins from different batches were used for each assay. Also, different concentrations of rLdMVK were used. To determine heat stability, the reaction mixture was incubated for 15 min at temperatures between 25 and 60°C, brought to room temperature, assay was performed and readings were taken. The temperature with the highest activity were considered as 100% and from this the residual kinase activity was determined.

#### Glycoprotein Staining

LdMVK protein was electrophoresed (SDS-PAGE) and staining of glycoprotein was carried out as stated in Pro-Q Emerald glycoprotein gel and blot stain kit manufacturer’s protocol (Molecular Probes, Eugene, Oreg). Protein in gel was fixed in a solution of 50% methanol and 5% acetic acid and washed in 3% acetic acid solution. Gel was incubated in oxidizing solution provided in the kit for 30 min and washed two times. Incubation in Pro-Q Emerald 300 staining solution was done for 2 h and stained gel was then visualized by illuminating in UV light in BioRad transilluminator. CandyCane™ glycoprotein molecular weight standards (Molecular Probes, Eugene, OR, USA) containing a mixture of alternate glycosylated and non-glycosylated proteins was separated by SDS-PAGE and stained by Pro-Q Emerald glycoprotein gel and blot stain kit, and it served as positive and negative control for the experiment. The staining was performed at least three times and protein from different batches were used each time.

#### Immunofluorescence Assay

Immunofluorescence assay was carried out to know the localization of MVK within parasite using anti-LdMVK antibody and anti-Ld-pyruvate phosphate dikinase antibody (glycosomal marker, raised in mice, 1:1,000 dilution). Briefly, exponentially grown *L. donovani* (5 × 10^6^ cells/ml) of Ag83 strain were collected by centrifugation, washed three times in PBS. Cells were incubated in fixation/permeabilization solution (BD Biosciences) for 30 min at 4˚C. Blocking (3% BSA in PBS) was performed for 30 min and cells were incubated for 45 min with anti-MVK antibody (1:200 dilution) prepared in 1% BSA and Triton X-100 containing PBS. Cells were then incubated with FITC conjugated anti-rabbit antibodies (1:200 dilution; Jackson ImmunoResearch; #AB_2337972) in dark for 45 min. Further, cells were incubated with anti-pyruvate phosphate dikinase antibody (1:200 dilution; glycosomal marker) for 30 min, following incubation with TRITC conjugated anti-mice antibodies (1:200 dilution; Jackson ImmunoResearch; #AB_2337972). Washing of cells were carried out with permeabilization/wash buffer (BD Biosciences; #554723) three times. Finally, cells were resuspended in PBS, mixed with prolong anti-fade solution (ThermoFisher Scientific; #P10144) and photographs were obtained from confocal laser scanning microscope (LSM880 zeiss) using a 100× Numerical aperture (NA) 1.44 PlanApo oil immersion objective. The assay was performed more than three times.

### Studies on Host Immune Response to MVK

#### Peripheral Blood Mononuclear Cells and Macrophages Isolation

To estimate the effect of r-LdMVK on immune response of host and parasite entry, venous blood from a non-endemic healthy control was collected in heparinized tube and mixed with phosphate buffer saline (PBS) (1:1). This suspension was layered on Ficoll-Hypaque (Sigma; #17144003) in 1:1 ratio and density gradient centrifugation was performed. Obtained peripheral blood mononuclear cells (PBMC) were washed thrice with Gibco™ RPMI-1640 medium (ThermoFisher Scientific; #31800022). For infection study, monocytes were segregated from PBMC in plastic culture flask since they have a property of plastic adherence. Five times washing with pre-warmed PBS was performed to remove non-adherent cells. Cell viability was evaluated by trypan blue dye (Ferreira et al., 2016). Adherent cells were cultured in complete RPMI-1640 medium (10% FBS) for up to 6 days and the medium was replaced after every 3 days. Accutase (Sigma; #A6964) was used to obtain adherent macrophages, cells were counted and re-adhered on chamber slides or six-well plate.

#### Enzyme Linked Immunosorbent Assay

The level of Interferon-γ (IFN-γ), Interleukin-12 (IL-12), Interleukin-2 (IL-2), Tumor necrosis factor-α (TNFα) in addition to Interleukin-10 (IL-10) and Interleukin-4 (IL-4) were calculated with commercial ELISA kits. PBMC’s from healthy person (1 × 10^6^ cells/ml) were plated in 24-well culture plates for 24 h. Soluble *Leishmania* antigen (50 ug/ml), r-LdMVK (1 ug/ml), or lipopolysaccharide (LPS) (100 ng/ml) (Sigma; #S4881) were added in triplicate wells, incubated for 16 h and cytokine response was observed according to the manufacturer’s protocol (BD OptEIA kit, USA). The results were obtained as picograms of cytokine/ml, based on the standard curves generated using a recombinant cytokine provided in the kit. Anti-inflammatory to pro-inflammatory cytokine ratios associated with different antigens-induced PBMC were compared. The experiment was carried out with three biological replicates, each performed in triplicates.

### Infection Studies

#### Cell Binding Assay

##### Method 1

PBMC-derived macrophages (5 × 10^4^) were seeded in 96-well microtiter plates and incubated overnight in RPMI 1640 supplemented with 10% FBS at 37°C in 5% CO_2._ Cells were fixed with 3.7% paraformaldehyde in PBS, washed three times with PBS, and blocked with 10%FBS diluted in PBS for 1 h at room temperature. Cells were washed three times with 0.05% Tween 20 containing PBS (T-PBS). Increasing amount of purified recombinant MVK was added to the wells (0.1 to 15 ug/ml) and incubated for 1 h at room temperature. Cells were washed with T-PBS and incubated with anti-MVK antibody (1:1,000 dilution) for 30 min and then with HRP tagged anti-rabbit antibody (1:1,000 dilution) for 30 min. After three washes with T-PBS, 3,3′,5,5′-Tetramethylbenzidine (TMB) (BD Biosciences; #555214) was added, reaction was stopped and absorbance was measured at 495 nm. There were three biological replicates under our study and for each condition ELISA were performed in triplicates.

##### Method 2

MVK binding to host cells was confirmed as previously described (Huynh and Carruthers, 2016). Precisely, r-LdMVK (1 µg/ml) or soluble *Leishmania* antigen was incubated with PBMC-derived macrophages adhered to six-well plate for 1 h. Monolayer cells were then washed with PBS-CM (1X PBS, 1 mMCaCl_2_, and 1 mM MgCl_2_) four times and the last wash was stored to check for r-LdMVK presence. Cells were lysed with RIPA buffer supplemented with protease inhibitor cocktail (Roche; #11697498001). The obtained cell bound fraction was separated by 12% SDS-PAGE and immunoblotted with anti-MVK antibody.

#### Generation of MVK-Overexpression Parasites

For the generation of MVK-OE (MVK-Overexpression) parasites, the LdMVK coding sequence was amplified using following primers: FP: 5’-TTTAAGCTTATGCCAAAGCCCGTCAAG-3’andRP: 5’-TTTTGGATCCCAGGTTTGACCCGGTGG-3’. The amplified products were ligated within HindIII and BamHI restriction sites and sense cloning was done in the same vector. The resulting recombinant pLGFPN vector was cloned in DH5α cells for stability. For transfection, late log phase promastigotes (2 × 10^8^ cells/ml) were washed with electroporation buffer containing 6 mM glucose, 21 mM 4-(2-hydroxyethyl)-1-piperazineethanesulfonic acid (HEPES), pH 7.5, 5 mM Potassium chloride (KCl), 137 mM NaCl, and 0.7 mM Disodium phosphate (Na_2_HPO_4_) and transfected with recombinant pLGFPN (10 µg) by electroporationin 4 mm electroporation cuvette using a Gene Pulsar (Bio-Rad). Electroporation was carried out according to the high-voltage protocol: 25 µF, 1,500 V (3.75 kV/cm) pausing 10 s between two pulses. Parasites were allowed to recover for 24 h and *Leishmania* that stably incorporated pLGFPN and pLGFPN-LdMVK vectors were chosen by culturing parasites for 4 weeks in the presence of increasing antibiotic Geneticin (G418) concentration (5 µg/ml to 50 µg/ml).

#### Quantitative PCR

Fourth-day culture of MVK-overexpressing and vector control strains were harvested at 800 g, 5 min and washed with PBS twice. Pellet was collected, resuspended, cells were counted, and 10^7^ cells were used for each strain. RNA was extracted from the cells using the TRIzol™ Plus RNA purification kit (ThermoFisher Scientific, #12183555) following manufacturer’s protocol. Amount of extracted RNA was quantified by Nanodrop Spectrophotometer. Exon of sequences were identified from the NCBI database and *MVK* and *18S rRNA* qPCR primers were designed using IDT (Integrated DNATechnologies, USA). MVK FP sequence was: 5’ CGGATGAAGAGGTGAATCAGAG 3’; and MVK RP sequence was: 5’GTATGGCGGACTCATTTCGTA 3’. *18S rRNA* was used for normalization and its FP and RP sequences were: 5’GGCCCTGTAATTGGAATGAGTC 3’; and MVK RP sequence was: 5’CCAAGATCCAACTACGAGCTT 3’ respectively. Quantitative Polymerase Chain Reaction (qPCR) conditions were: 30 s at 95°C, 45 s at 58°C, 30 s at 72°C (45 cycles), and 5 min at 95°C for initial denaturation. cDNA was prepared using High capacity cDNA reverse transcription kit (ThermoFisher Scientific, #4368814) and qPCR was performed using SYBR green PCR master mix (ThermoFisher Scientific, #4309155). Negative template controls were made for each qPCR analysis. Three biological replicates were used and each time the reaction was carried out in triplicates.

#### Infection of Macrophages

PBMC-derived macrophages (5 × 10^4^ cells/well) were prepared as previously described, seeded onto four-well chamber slides (Nunc Lab-Tek) and incubated overnight in RPMI 1640 supplemented with 10% FBS at 37°C in 5% CO_2._ Cells were washed with PBS and infected with metacyclic *L. donovani* promastigotes in 0.4 ml RPMI 1640 at 1:10 ratio (macrophage: parasite). After 4 h at 37°C in 5% CO_2_, parasites were removed by three PBS washes and chamber slides were incubated for required periods of time. This was followed by methanol fixation and either Giemsa staining or immunofluorescence. For MVK treatment, r-LdMVK (1 µg/ml) was added to the macrophages along with the parasites. For antibody inhibition assay, promastigotes were pre-incubated with anti-MVK antibody for 30 min at 25°C. The measurement of intracellular parasites was carried out in 400 macrophages per well and the data was shown as the total number of intracellular parasites per 100 macrophages. Infection was repeated with mutant strains of parasites: MVK-Overexpression/Vector control strains (4 h duration) as already discussed. For each condition, experiment was carried out in triplicates and the data were analyzed using an unpaired Student’s *t* test and indicated as mean ± SE of three independent experiments. P value <0.05 was considered to be significant.

### Phosphoprotein Assay

PBMC-derived macrophages (4 × 10^6^) were seeded and grown for 24 h. Cells were incubated for another 24 h with serum-free RPMI to reduce constitutive signaling. Following starvation, cells were incubated with r-LdMVK (1 ug/ml) from 5 to 90 min. Cells were then washed with PBS containing 2 mM sodium orthovandate (Na_3_VO_4_) and 5 mM sodium fluoride (NaF) to minimize phosphatase activity. Cell lysis was performed with mammalian lysis buffer (Cell Signaling Technology; #9803) supplemented with phosphatase and protease inhibitors. Protein was quantified by Bicinchonic acid method and probed with antibodies against Phospho-Extracellular signal-regulated kinase (ERK-1/2) (1:1,000 dilution; Cell Signaling Technology, #9101), ERK-1/2 (1:1,000 dilution; Cell Signaling Technology, #9102), Phospho-p38 MAP kinase (1:1,000 dilution; Cell Signaling Technology, #9211), p38 MAP kinase (1:1,000 dilution; Cell Signaling Technology, #9212), Phospho-Cortactin (1:1,000 dilution; Merck, # AB3795), Cortactin (1:1,000 dilution; Cell Signaling Technology, #3502), and house-keeping gene, Glyceraldehyde-3-phosphate dehydrogenase (GAPDH) (1:1m000 dilution; Santa Cruz Biotechnology, #sc-32233). Each experiment was carried out three times.

### Statistical Analysis

Statistical analysis was carried out in GraphPad Prism (GraphPad Software, Version 6.0). Results are expressed as mean ± SE of three or more independent experiments (Soares-Silva et al., 2016). Studies with three or more groups were examined by one-way ANOVA and Tukey’s *post hoc* test. Experiments with two groups were studied by unpaired student’s t-test. P-values of 0.05 or lesser were considered significant.

## Results

### *L. donovani* Expresses and Releases Mevalonate Kinase Enzyme

**Figure 1:**
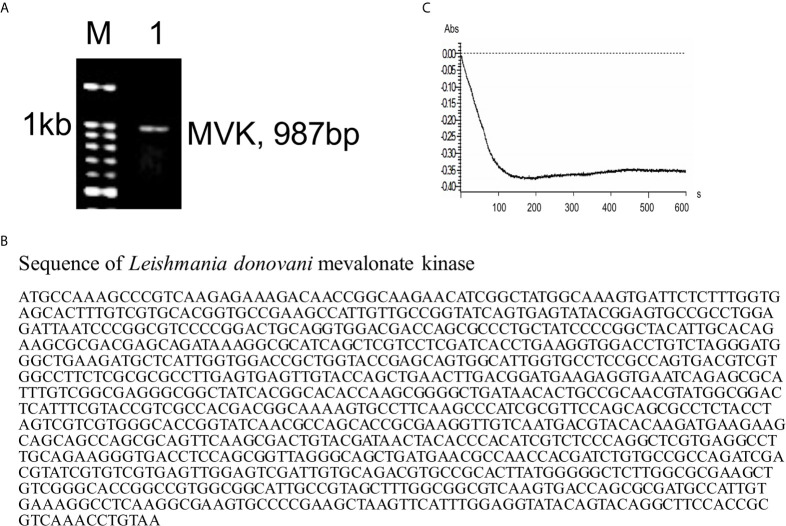
Presence of mevalonate kinase in *L. donovani* and its release in extracellular media. **(A)** PCR amplification was carried out using *MVK* gene specific primers and *L. donovani* Ag83 strain genomic DNA as template to reveal its presence in the parasite. Kb, kilobase; M, DNA ladder; 1, MVK-PCR product. Similar results were obtained from at least two independent experiments. **(B)** The PCR fragment amplified using *MVK gene* specific primers was sequenced using di-deoxy sequencing and full length *MVK* gene sequence was obtained. **(C)** Parasite was incubated in serum-free medium for 6 h at 37°C and culture supernatant was collected and concentrated. The concentrated supernatant consisting of released proteins was checked for the presence of MVK by mevalonate kinase assay. Decrease in absorbance due to NADH oxidation relates to increased MVK activity. Representative image of at least three independent experiments is shown. Blank assays were performed with centricon concentrated incomplete Medium199.

The presence and function of MVK protein has not been reported in *L. donovani*. TriTrypDB database was explored and revealed MVK location on chromosome 31. Sequence of the putative LdMVK gene was obtained from the NCBI database (LDBPK_310580). PCR amplification from the genomic DNA of Ag83 strain clearly showed the presence of MVK (**Figure 1A**). Sequencing of the PCR fragment showed 98% similarity with the putative MVK sequence obtained from NCBI database (**Figure 1B**). Secretion prediction was performed using SecretomeP 2.0 Server (Bendtsen et al., 2004) and SecP score of 0.718 was obtained with recommended thresholds, 0.5 for bacterial sequences and 0.6 for mammalian sequences. The protein was predicted to be a non-classically secreted protein and devoid of any signal sequence. Next, MVK activity was checked in culture supernatant. *L. donovani* promastigotes were incubated in serum-free Medium 199 for 6 h at 37°C, culture supernatant was collected and concentrated. The concentrated medium containing *Leishmania* secretory proteins was added to the reaction mixture described in the methods section to start the reaction and O.D. at 600 nm was taken that showed mevalonate kinase activity (**Figure 1C**). For blank assays concentrated serum free M199 was used. This indicated that MVK is released in the outer medium by *L. donovani* promastigotes.

### Expression and Characterization of Recombinant Mevalonate Kinase

**Figure 2:**
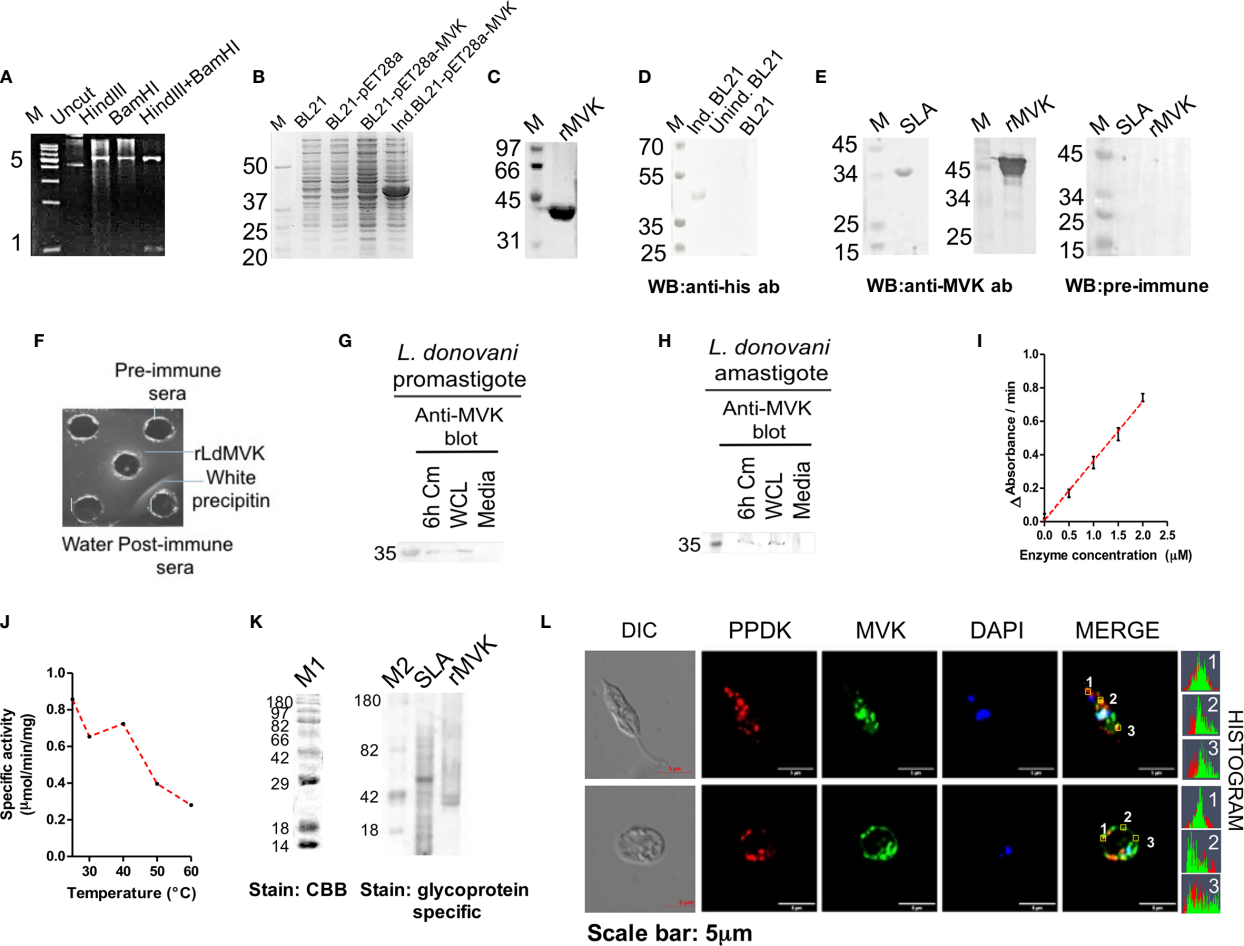
Characterization of *L. donovani* mevalonate kinase. **(A)** Figure showing confirmation of cloning after HindIII+BamHI double digestion of rpET28a-MVK. HindIII+BamHI double digested product was run on agarose gel electrophoresis. M, DNA ladder; uncut, uncut pET28a-MVK; HindIII, HindIII digested recombinant plasmid; BamHI, BamHI digested recombinant plasmid; HindIII+BamHI, double digested HindIII+BamHI rpET28a-MVKproduct. Representative of at least three independent experiments is shown. **(B)** SDS-PAGE showing the expression of rLdMVK by induced and transformed BL21 cells. M, Protein marker; BL21, bacterial cell lysate; BL21-pET28a, pET28a transformed bacterial lysate; BL21-pET28a-MVK, pET28a-MVK transformed bacterial lysate; Ind. BL21-pET28a-MVK, pET28a-MVK transformed and induced bacterial lysate. **(C)** Ni-NTA purified sample stained with Coomassie brilliant blue. This purified r-LdMVK was used for all functional characterization experiments. rMVK: Sample from final purification step of Ni-NTA purified extract. **(D)** Western blot confirming the expression of rMVK. MVK expression by induced and transformed BL21(DE3) cells was verified by western blot of transformed cell lysate using anti-his antibody. Ind. BL21, transformed induced bacterial extracts expressing r-LdMVK; unind. BL21, transformed uninduced bacterial lysate; BL21, untransformed BL21 cells. **(E)** Validation of generated anti-MVK antisera. SLA and rMVK were probed with anti-MVK antisera; and pre-immune sera. M, protein marker; SLA, soluble *Leishmania* antigen; rMVK, recombinant mevalonate kinase; WB, western blot. **(F)** Oucherlony test validating anti-MVK antisera. Line of precipitation denotes the line where rMVK and anti-MVK antisera meet and interacts. Line of precipitation was not observed with pre-immune sera. **(G)** Western blot analysis of promastigotes conditioned medium (Cm) confirming secretion of MVK. Stationary phase promastigotes were washed three times in PBS, incubated for 6 h in serum-free M199 medium (1 × 10^8^ parasites/ml), culture supernatant was concentrated 25 times using 10MWCO centricon, and the 6 h conditioned medium was loaded on SDS-PAGE gel. Protein transferred on PVDF membrane was probed with anti-MVK antibody at 1:1,000 dilution. 6 h Cm, Ld promastigotes 6 h conditioned medium; WCL, 3% whole cell lysate of promastigotes; Media, concentrated serum-free M199 medium. **(H)** Western blot analysis of amastigotes conditioned medium (Cm) confirming secretion of MVK. Stationary phase axenic amastigotes were incubated for 6 h in serum-free M199 medium (1 × 10^8^ parasites/ml), culture supernatant was concentrated 25 times by 10MWCO centricon, and the 6 h conditioned medium was loaded on SDS-PAGE gel. Protein transferred to PVDF membrane was examined with anti-MVK antibody Fat 1:1,000 dilution. 6 h Cm, Ld promastigotes 6 h conditioned medium; WCL, 3% whole cell lysate of promastigotes; Media, concentrated serum-free M199 medium. Similar results were obtained from two biological replicates and images are illustrative of three or more unrelated experiments. **(I)** Specific activity of rLdMVK was determined with increasing MVK concentrations. Graph depicts a linear increase in MVK enzymatic activity with increasing concentration of rLdMVK. **(J)** Graph depicting a decline in enzymatic activity of rLdMVK on treatment at different temperatures (25 to 60°C). **(K)** Candycane^TM^ glycoprotein molecular weight standards (M1 and M2) were run on 12% SDS PAGE. CandyCane^TM^ glycoprotein molecular weight standards (Molecular Probes, Eugene, OR, USA) containing a mixture of alternate glycosylated and non-glycosylated proteins was separated by SDS-PAGE and stained by Pro-Q Emerald glycoprotein gel and blot stain kit, and it served as positive and negative control for the experiment. Staining with Coomassie Brilliant blue reveals all eight band; glycoprotein staining shows four glycosylated proteins bands. rLdMVK was run on 12% SDS-PAGE, oxidized using perchloric acid, and stained for carbohydrates (Emerald 300 Q glycoprotein stain); M1, Coomassie brilliant blue stained Candycane^TM^ glycoprotein marker; M2, glycoprotein stained Candycane^TM^ glycoprotein marker; SLA, soluble *Leishmania* antigen; rMVK, recombinant LdMVK; CBB, Coomassie Brilliant blue. **(L)** LdMVK is co-localized with glycosomal marker, PPDK. Double immunofluorescence pictures taken using confocal microscope depicts that LdMVK is present in glycosomes of *L. donovani* promastigotes and glycosomes, membrane and nucleus of *L. donovani* amastigotes. Differential interference contrast (DIC); DAPI (blue); rabbit anti-LdMVK (green); rabbit anti-LmPPDK (red); merged image (merged); histogram: overlapping histogram of PPDK and MVK shows co-localization of both the protein. Images are representative of three independent experiments.

Full length amino acid sequence of LdMVK has a molecular mass of 35.61 kD and a theoretical isoelectric point of 9.13 as predicted by Expasy. Protein sequence similarity between human MVK and *L. donovani* MVK was only 26%. LdMVK was cloned into *E. coli* (**Figure 2A**) and confirmed by DNA sequencing that showed 99% homology with putative MVK sequence. Recombinant protein (r-LdMVK) tagged with poly-histidine was expressed (**Figure 2B**) and MVK expression was confirmed through western blot technique using anti-his antibody (**Figure 2D**). The expressed protein was purified using Ni-NTA affinity chromatography and a single band of expected size was obtained (**Figure 2C**). The purified r-MVK was then used for polyclonal antibody production. Obtained anti-MVK antisera was validated for its specificity by western blot (**Figure 2E**) and Ouchterlony test (**Figure 2F**). Western blot of whole cell lysate (WCL) of *L. donovani* promastigotes and axenic amastigotes probed with anti-MVK antibody demonstrated a single band of expected molecular weight (35 kD) that further confirmed the presence of MVK in both forms of *L. donovani* (**Figures 2G, H**). The presence of MVK in the culture supernatant was confirmed by western blot. Both promastigotes and axenic amastigotes of Ag83 strain released MVK into the extracellular medium (**Figures 2G, H**). The size of secreted LdMVK was found to be the same as that of the cytosolic LdMVK, hence, the released forms are not proteolytically processed. WCL (whole cell lysate) was prepared from only 3% of the pellet and loaded. Supernatants can be compared to the diluted lysates of cell (3% of pellet). Natural death during the course of *Leishmania* culture does not include the release of intracellular content. It is earlier reported that glycosylation inhibitors inhibit Leishmanial infectivity (Nolan and Farrell, 1985; Kink and Chang, 1987), so we also attempted to verify if LdMVK is a glycoprotein using glycoprotein specific staining procedure as discussed in methods section. Glycoprotein specific staining successfully stained r-MVK confirming its glycoprotein nature (**Figure 2K**). Also, analysis of LdMVK model indicated the presence of three possible N-linked glycosylation regions and one O-linked glycosylation regions (**Supplementary Figure 1**).

Enzymatic activity of r-LdMVK was measured by methods described earlier (Ferreira et al., 2016; Duarte et al., 2018); by relating ADP release with oxidation of NADH by pyruvate kinase and lactate dehydrogenase. With increasing concentration of MVK, change in absorbance with respect to time increased linearly (**Figure 2I**). The specific activity of r-LdMVK was found to be 0.83 micromoles NADH/min/mg. Thermal stability was also studied and r-LdMVK was functional over broad range of temperature: 25–60°C and exhibited 33% of its optimal activity at 60°C. It was found to be temperature resistant as it retains its activity at 60°C (**Figure 2J**). All the enzymatic activity studies were carried out in glycine buffer. These results represent first step toward understanding of properties of MVK in *Leishmania*.

### Subcellular Localization of MVK in Promastigote and Amastigote Forms

To check the localization of MVK in both promastigotes and amastigotes, confocal laser scanning microscope (LSM880 zeiss) was used. Immunofluorescence detection of MVK probed with FITC-conjugated secondary antibody (1:200 dilution; Jackson ImmunoResearch; #AB_2337972) showed a glycosome-like punctuate pattern which co-localized with glycosome specific pyruvate phosphate dikinase in promastigotes (Bringaud et al., 1998) which was probed with TRITC-conjugated anti-mice antibodies (1:200 dilution; Jackson ImmunoResearch; #AB_2337972). In amastigotes, MVK was observed to be present in glycosome, nucleus as well as concentrated on the cell membrane (**Figure 2L**). Three spots selected on promastigote and amastigote were shown to have overlapping histogram of fluorescent signal from PPDK and MVK. This showed partial co-localization of MVK and PPDK.

### MVK Secretion Is Time, Temperature, and pH Dependent

**Figure 3:**
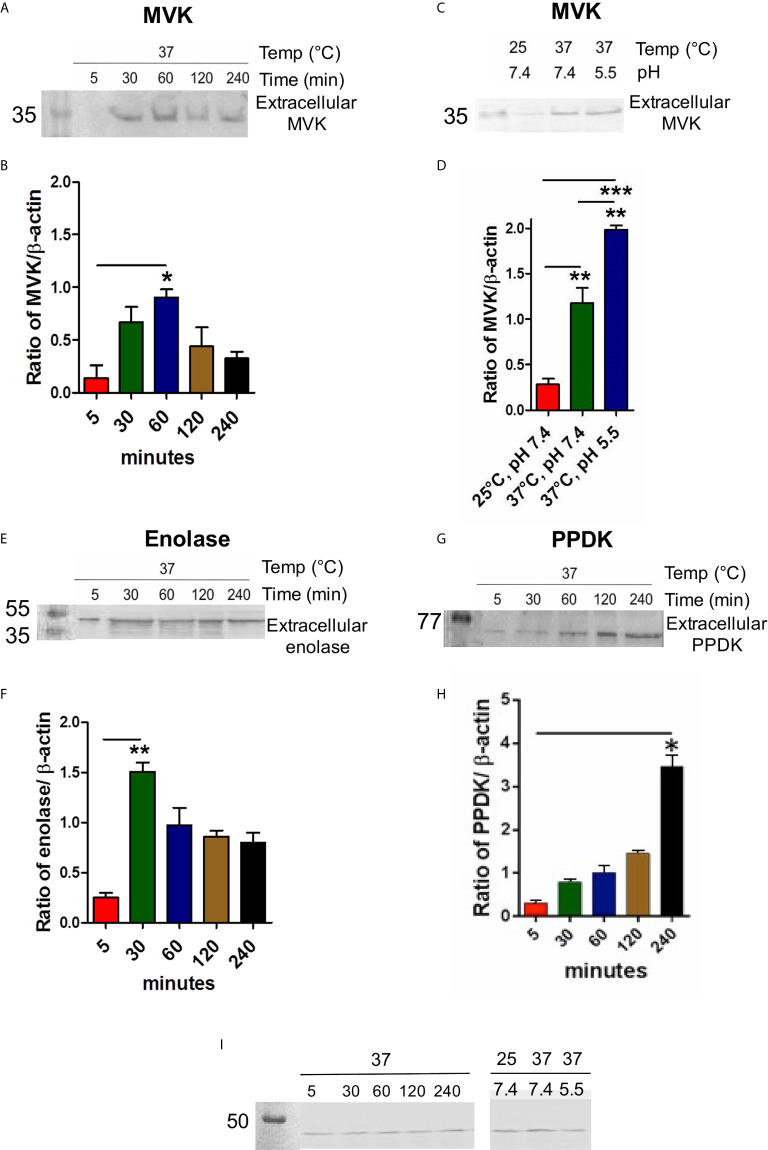
MVK secretion in *L. donovani* promastigotes is time, temperature, and pH dependent. Western blot of promastigote conditioned medium collected at different time points or different environment conditions and normalized with housekeeping gene (*L. donovani* actin). Stationary phase parasites (1 × 10^9^cells) were washed three times in PBS and resuspended in pre-warmed serum free medium to final density of 1 × 10^8^ cells/ml. Parasites were stimulated for secretion at 37°C for 5 to 240 min. Conditioned medium consisting of extracellular proteins were probed with anti-MVK antibody **(A)**, anti-enolase antibody **(E)**, anti-PPDK antibody **(G)**, and compared. Parasite lysate for each sample was probed with anti-Ld actin antibody that served as cell number control. Graphical representation of densitometry values of extracellular MVK / intracellular β-actin **(B)**, extracellular enolase / intracellular β-actin **(F)**, extracellular PPDK / intracellular β-actin **(H)** with respect to time. **(C)** Release of MVK was observed at different temperatures and pH conditions: 25°C and 7.4 pH; 37°C and 7.4 pH; and 37°C and 5.5 pH. Serum-free M199 medium was maintained at different temperatures and pH and parasites were incubated in it for 1 (h) Conditioned medium was obtained, concentrated, and western blot was performed using anti-MVK antibody. **(D)** Graphical representation of densitometry values of extracellular MVK obtained from different conditions normalized with β-actin. **(I)** 3% whole cell lysates from all the conditions were probed with anti-actin antibody. Error bars in all graphs represent standard error from three independent experiments performed in triplicates. p value < 0.05 is denoted by *, p value ≤ 0.01 is denoted by ** and p value ≤ 0.001 is denoted by ***.

The exoproteome of parasite was examined for the level of MVK release after temperature stimulation (37°C) to mimic initial infection condition. Sixty min post heat stress (37°C), maximum amount of MVK was observed in the extracellular medium, which declined with time (**Figures 3A, B**), suggesting its probable involvement in initial stage of infection. To examine if the release pattern is related to protein function, time kinetics of enolase release was studied. *Leishmania* enolase facilitates invasiveness (Vanegas et al., 2007) and its release was maximum at 30 min post temperature stimulation which declined with time (**Figures 3E, F**). On the contrary, the release of pyruvate phosphate dikinase (PPDK), a gluconeogenesis pathway enzyme, increased linearly with time (**Figures 3G, H**). To our knowledge, this is the first report on the release of PPDK by *L. donovani*.

*Leishmania* encounters heat shock and acidic environment as they enter mammalian host (37°C) through sandfly bite (26°C). Therefore, it was of interest to examine changes in release of MVK in response to such stimulus. Conditioned medium from *L. donovani* promastigotes incubated at different conditions were collected after 1 h incubation. Temperature stimulation at 37°C induced the release of MVK by ~4 times compared to parasites incubated at 25°C (**Figures 3C, D**). On the other hand, phagolysosome-like conditions (37°C and pH 5.5) increased the secretion by ~6.5 times in comparison to MVK secretion at 25°C (**Figures 3C, D**). For these experiments, intracellular actin was used as control to ensure that the exoproteome was from equivalent number of cells (**Figure 3I**). Each sample was normalized against ß-actin of parasite lysate.

### r-LdMVK Stimulates PBMCs to Express Th2 Cytokine Profile

**Figure 4:**
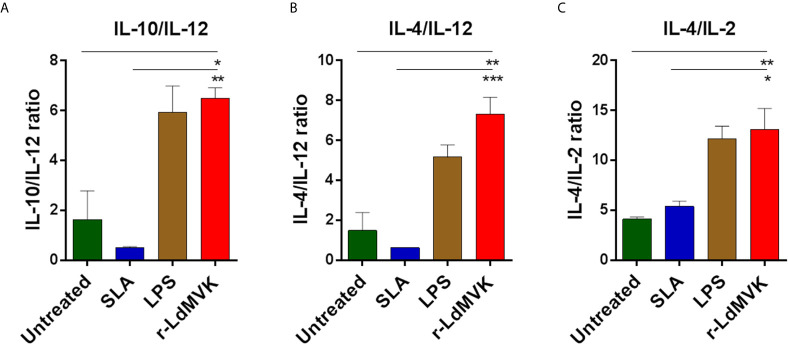
Secreted level of cytokines in macrophages after r-LdMVK stimulation. **(A)** IL-10/ IL-12, **(B)** IL-4/ IL-12, and **(C)** IL-4/ IL-2 cytokine ratio of PBMC of healthy control, cultured in the presence of r-LdMVK, soluble *Leishmania* antigen (SLA), or lipopolysaccharide (LPS). PBMC was assessed for their effect on various cytokines release: IFNγ, IL-12p70, TNF⍺, IL-10, IL-2, and IL-4. Levels of cytokines were measured by ELISA after 16 h treatment. Unstimulated culture was used as a control. The data represents mean±SE of three independent biological replicates (including three technical replicates). p value < 0.05 is denoted by *, p value ≤ 0.01 is denoted by ** and p value ≤ 0.001 is denoted by ***.

*Leishmania* manipulates host defense system for their own survival and targeting these immune components is a reliable method to monitor the disease. In the same queue we studied the potential of LdMVK in shifting immune axis in support of parasite survival and disease progression (**Table 1**). We have observed that the IL-10/IL-12 ratio was 6.4 ± 0.41 in the LdMVK treated PBMC, 0.5 ± 0.02 in soluble *Leishmania* antigens (SLA) treated PBMC, and 1.6 ± 1.1 in untreated PBMC obtained from healthy person (**Figure 4A**). Further, IL-4/IL-12 ratio was found to be 7.33 ± 0.8 in LdMVK treated cells, 0.6 ± 0.01 in SLA treated cells, and 1.4 ± 0.9 in untreated cells of a healthy person (**Figure 4B**). IL-4/IL-2 cytokine ratio in LdMVK treated cells was found to be 12.2 ± 2.6 compared to 4 ± 0.2 in untreated cells of healthy person (**Figure 4C**). These results suggest that MVK has an immunosuppressive function.

**Table 1:** Secreted cytokine values (pg/ml) of PBMC treated with r-LdMVK, soluble *Leishmania* antigen (SLA), or lipopolysaccharide (LPS).

	IFNγ	IL-12	TNF⍺	IL-2	IL-10	IL-4
UN	112 ± 18	41 ± 8	69 ± 7	31 ± 1	126 ± 32	128 ± 4
SLA	310 ± 19	96 ± 21	81 ± 24	34 ± 5	152 ± 13	182 ± 9
LPS	109 ± 46	33 ± 9	161 ± 14	4 ± 4	195 ± 35	165 ± 21
MVK	155 ± 61	29 ± 5	108 ± 5	18 ± 5	193 ± 44	213 ± 26

Unstimulated culture was used as a control.

### LdMVK Has a Role in Phagocytosis

**Figure 5:**
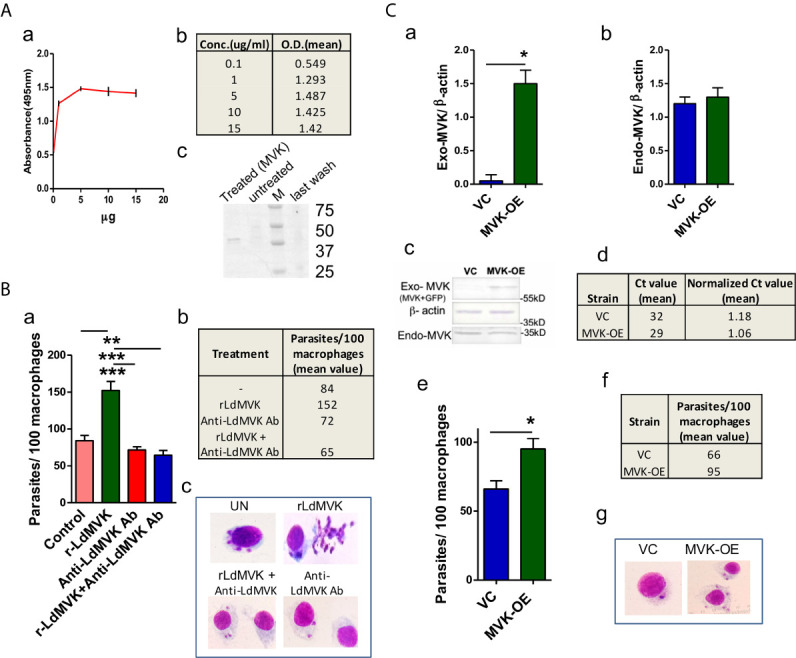
LdMVK facilitates parasite invasion. **(A)** rMVK binds to the surface of macrophages. (a) Graph depicts the binding of rLdMVK on macrophages in a dose-dependent manner. Fixed macrophage cells were incubated with increasing protein concentration. The result shows mean of three independent experiments performed in triplicates. (b) Table representing the mean O.D. of experiments performed in triplicates of aforesaid described adhesion assay. (c) Blot representing binding of r-LdMVK to macrophage cell surface. PBMC-derived macrophage was incubated with r-LdMVK for 1 h, washed four times with PBS-CM, and lysed using RIPA buffer containing phosphatase inhibitors. Lysate was separated on SDS-PAGE gel and incubated in anti-MVK antibody. The image is representative of three independent experiments. Treated, macrophages incubated with r-LdMVK; untreated, macrophages without any protein incubation; M, protein marker; last wash, last wash of three washes performed post MVK incubation. **(B)** Infection of macrophages on rLdMVK treatment. (a) PBMC-derived macrophages were treated with Ld promastigotes, 1 µg/ml r-LdMVK+Ld promastigotes, anti-MVK antibody + Ld promastigotes and r-LdMVK + anti-MVK antibody + Ld promastigotes for 4 (h) Parasite to cell ratio was 10:1. Error bars shows SE from three independent studies. (b) Table depicting the mean value of parasites internalized per 100 macrophages on different incubations. (c) Representative image of Giemsa stained infected macrophages on various treatments. **(C)** Infection of macrophages by MVK-overexpressed and vector control parasites. (a) Bar graph representing normalized band intensity of exogenous MVK (exogenous-MVK/ β-actin) in VC and MVK-OE parasites. Error bars shows SE from three independent studies. (b) Bar graph representing no significant change in the level of normalized band intensity of endogenous MVK (endogenous-MVK/ β-actin) in VC and MVK-OE parasites. Error bars shows SE from three independent studies. (c) Western blot confirmation of generation of MVK-Overexpression strain. Whole cell lysate of MVK-Overexpression and vector control strains of Ag83 parasites were prepared, separated on SDS-PAGE gel, and probed with anti-MVK antibody. The image is representative of three independent experiments. VC, only pLGFPN transfected parasites; MVK-OE, GFP-tagged MVK-overexpressed parasites. (d) Real time PCR confirmation of generation of MVK-Overexpression strain. RNA from MVK-Overexpression and vector control strains of Ag83 parasites were prepared; after cDNA preparation real time PCR was carried out. Table is showing the mean normalized Ct value from these experiments. (e) Bar graph representing the number of internalized parasites per 100 macrophages. PBMC-derived macrophages were treated with mutant stains of parasites with 1:10 macrophage to parasite ratio for 4 (h) Macrophages were washed, stained, and internalized parasites were counted. The data represents the mean±SE of three experiments carried out in triplicates. (f) Table showing the mean value of internalized parasites per 100 macrophages from three experiments. (g) Representative image of Giemsa stained vector control and MVK overexpressed parasites treated macrophages. p value < 0.05 is denoted by *, p value ≤ 0.01 is denoted by ** and p value ≤ 0.001 is denoted by ***.

Cell membrane adhesion assay using r-LdMVK and fixed PBMC-derived macrophages showed an increase in enzyme bound to cell surface of macrophage with increasing concentration up to 1 µg/ml (**Figures 5Aa, b**). No increase in bound enzyme was observed on increasing the concentration further. Hence this concentration of protein was used later in experiments. To validate the association of r-MVK with macrophage, r-MVK was incubated with PBMC-derived macrophages for 1 hr and cell lysate was immunoblotted against anti-LdMVK antibody. LdMVK specific staining was observed in the macrophages confirming the association of LdMVK with host macrophages (**Figure 5Ac**). As discussed earlier, maximum MVK is released 1 h post temperature stimulation (**Figures 3A, B**), when most of the parasite enters macrophage. This led us to speculate that MVK may have a role in parasite internalization. PBMC-derived macrophages from healthy person were treated with 1 µg/ml r-LdMVK followed by incubation with promastigotes (Ag83 strain) for 4 h. It was observed that LdMVK treatment increased phagocytosis by ~1.8-fold (**Figure 5B**). Antibody inhibition using anti-LdMVK antibody prevented MVK induced parasite entry, affirming that it has a role in phagocytosis. For validation, we generated MVK-Overexpression and Vector control strains of *L. donovani* parasites (**Figure 5C**). Western blot of MVK-OE and VC parasite’s lysate with anti-MVK antibody showed two bands indicating that exogenous protein (GFP and MVK-GFP) was expressed. MVK was overexpressed by ~2.3-fold in MVK-OE parasites as shown by western blot experiments (**Figure 5Cc**). qPCR results indicated mean normalized Ct value of 1.06 and 1.18 for MVK-overexpressed and vector control strains respectively and a difference in mean Ct value of 3 (**Figure 5Cd**). Infection assay wherein PBMC-derived macrophages were treated with mutant strains of parasites for 4 h, demonstrated significant increase in number of internalized parasites (~1.4-fold) in MVK-OE strain compared to VC strain (**Figure 5Ce**).

### LdMVK Treatment Leads to Host Cell Signaling Proteins’ Phosphorylation

**Figure 6:**
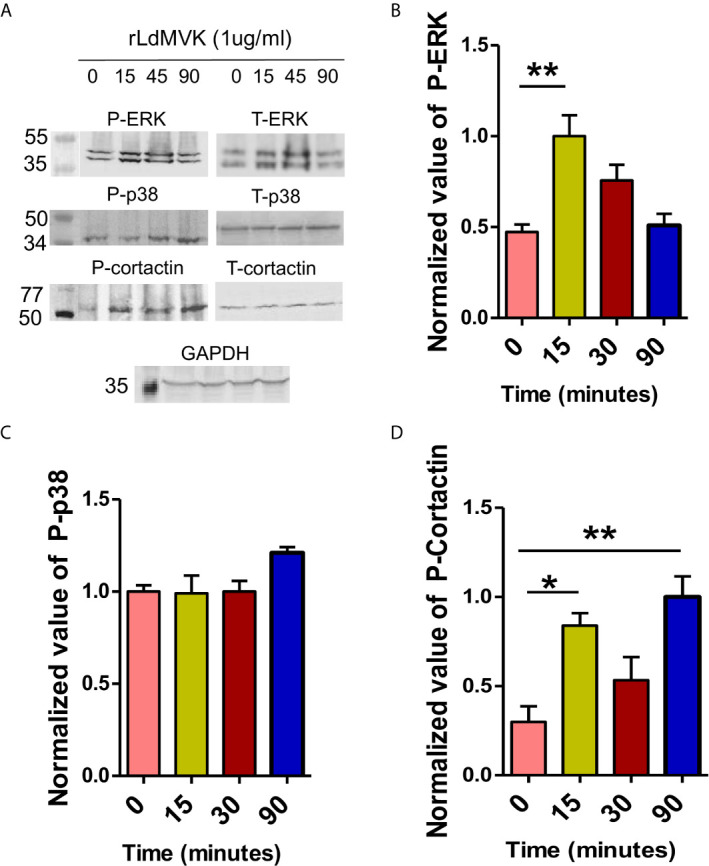
LdMVK induces the phosphorylation of RAW 264.7 cell signaling proteins. **(A)** Western blot of rLdMVK induced time dependent phosphorylation of RAW cell lysate probed with Phospho-ERK-1/2, ERK-1/2, Phospho-p38 MAP kinase, p-38 MAP kinase, Phospho-cortactin, cortactin, and GAPDH. **(B)** Bar graph representing normalized densitometry values of rLdMVK induced time-dependent phosphorylation of ERK-1/2, with maximum phosphorylation at 15 min. **(C)** Cortactin shows increased LdMVK-dependent phosphorylation at 15 and 90 min post-induction. **(D)** Phospho-p38 did not show significant activation on MVK-induction. 0 min studies served as negative control of experiment, i.e., RAW macrophages without LdMVK incubation. Anti-GAPDH was used as loading control in all the experiments. Band densitometry was performed using ImageJ software. Data are represented as the mean ± SEM of three experiments and blots are representative image of those. For total ERK-1/2, total-p38, and total Cortactin blots, the membrane was stripped and re-probed. p value < 0.05 is denoted by *, p value ≤ 0.01 is denoted by **.

To understand the mechanism behind the increased parasite entry and immunomodulation induced by LdMVK, its effect on phosphorylation status of key host cell signaling components was observed with respect to time (0 to 90 min). Phosphorylated ERK 1/2 was shown to be upregulated more than two times after 15 min MVK treatment (**Figures 6A, B**). On the contrary, no significant change was observed in the phosphorylation status of p38 MAP kinase (**Figures 6A, C**). Cortactin, a key actin scaffold protein, was phosphorylated three times more at 90 min on MVK treatment (**Figures 6A, D**).

## Discussion

The characterization and role of individual secretory proteins of *Leishmania* has remained elusive since many years. Here we describe first time one such novel protein from *L. donovani*, the mevalonate kinase (MVK) in *Leishmania* infection. Our investigation demonstrated that LdMVK is secreted by the parasite during *in vitro* cultivation and plays an important role in VL pathogenesis by assisting in the initial phase of infection and contributing to the efficient entry of the parasites in the host cells. Our results also suggest that LdMVK is capable of inducing interleukin-4 and interleukin-10 secretion through subversion of host cell. Our work sheds some light on the role of parasite in the entry process which is thought to largely dependent on macrophage in *Leishmania* infection.

The present study confirmed the presence of MVK protein in both promastigote and axenic amastigote of *L. donovani*. Cloned, expressed, and purified recombinant LdMVK protein was found to be functional (0.83 µmol/min/mg), as assessed by *in vitro* assays using its synthetic substrate, mevalonic acid. On the contrary, *L. major* MVK activity was found to be significantly low (20 pmol/min/mg) in another study (Sgraja et al., 2007). LdMVK modelled structure is predicted to have similar α/βfolding patterns as that of LmMVK. The reliability of the proposed model was also supported by the presence of both N- and O-glycosylation sites. The glycosylation sites that were predicted, recommended that glycans will fit into these sites without steric clashes, thus signifying LdMVK glycosylation probability. Further, r-LdMVK expressed in a bacterial system was studied and revealed the glycosylated nature of protein. It is now established that bacteria express glycoproteins and carry out both N-linked and O-linked glycosylation pathways with many commonalities with their eukaryotic counterpart (Nothaft and Szymanski, 2010) and both the *N*-linked and *O*-linked glycosylation pathways can modify multiple proteins. Considering the significance of glycosylation in *Leishmanial* virulence and extracellular vesicle pathophysiology (Kielian et al., 1982; Wang et al., 1996; Escrevente et al., 2011; Staubach et al., 2012; Gerlach et al., 2013), it was thought that the glycosylation of MVK might assist it in performing cellular functions.

Partial co-localization of MVK with the glycosomal protein, PPDK in typical punctuate structures showed its glycosomal compartmentalization. This was in compliance with the previous studies that showed MVK in *Leishmania donovani* is glycosome residing (Jardim et al., 2018). Other work done in *L. major*, *T. cruzi*, and *T. brucei* also displayed colocalization with glycosomes (Carrero-Lerida et al., 2009; Ferreira et al., 2016). The compartmentation of environment-sensitive parts of metabolism, like MVK, within glycosome could help parasites to acclimatize to acidic pH in phagolysosome. In contrast to promastigotes, it was observed that MVK in amastigotes were concentrated at the periphery of the cells, indicating that probably secretion of MVK in amastigotes is higher than that in promastigotes. Similar result was observed while mimicking phagolysosome-like condition that led to significant increase in MVK secretion. However, this was not confirmed in our study.

Silverman et al. have suggested the release of whole glycosome or glycosomal cargo by parasites outside cells (Silverman et al., 2008). Also, metabolic enzymes are shown to be post-translationally modified, changing their location and allowing to phosphorylate protein substrates. MVK’s glycosylated nature, specific compartmentalization in glycosome, and its secretory nature in *Trypanosoma cruzi* (Kielian et al., 1982; Wang et al., 1996; Ferreira et al., 2016) embarked us to investigate if it is secreted by *L. donovani* too. Going through the published secretome data we found that mevalonate kinase protein is not listed in the *L. donovani* and *L. infantum* secretome profiles (Silverman et al., 2008; Douanne et al., 2020). But, the list also does not include some other known *Leishmania* secreted proteins like serine protease, histidine acid phosphatase, and glycoprotein 63 (Jaffe and Dwyer, 2003; Joshi et al., 2004; Choudhary et al., 2010a). This could be because of the stringency in data collection method to involve abundantly secreted proteins.

The secretion of MVK into extracellular medium was confirmed by mevalonate kinase enzymatic assay and western blot of culture supernatant obtained from both promastigote and axenic amastigote form. Obtaining amastigotes from infected macrophages led to death of more 5% of amastigotes. Death of amastigotes due to harsh processing conditions could release their intracellular contents outside the cell interfering with the secretory protein profile. Hence, axenic amastigotes were used in all the experiments. The size of secreted LdMVK was found to be the same as that of the cytosolic LdMVK, suggesting that the released forms are not proteolytically processed as in the case of MIC2 (Carruthers et al., 2000), MIC5 (Brydges et al., 2000), and glycosylphosphatidylinositol-anchored micronemal antigen (TgGAMA) (Huynh and Carruthers, 2016). It is well known that eukaryotes use classical secretion pathway for the distribution of proteins throughout cell and outside the cell. But in *Leishmania*, most reports assert that majority of secretory proteins lack a signal peptide (Silverman et al., 2008; Kima et al., 2010; Hassani et al., 2011). Though the secretory pathway in this parasite is not well studied, present understanding exhibit non-classically secreted modes: exosome, apoptotic bodies, and plasma membrane blebs, all involving microvesicles (Silverman et al., 2010; Hassani et al., 2011; Forrest et al., 2020). The absence of signal sequence in MVK and prediction as non-classically secreted protein by Secretome P 2.0 server, suggested that its release is by non-classical secretion pathway.

Most of the *L. donovani* promastigotes are internalized by 60 min (Chakrabarty et al., 1996). Maximum release of LdMVK, 60 min after heat treatment could be because of its involvement in initial phase of infection (host cell colonization). *L. donovani* enolase, that supports parasite entry in host cell also shows similar release pattern in our study, whereas the release of PPDK (unknown secondary function) was shown to increase with time. This is one of the few studies that described the dynamics of protein release with respect to time. It indicated that the amount of protein released outside cell is not just dependent on the environment but also on the time of infection. If the maximum release time of a protein is dependent on its function is yet to be studied.

It is known that *Leishmania* protein release involves functional enrichments based on temperature and pH variations (Silverman et al., 2010; Hassani et al., 2011). Heat shock for 24 h increase vesicle release by 3-fold and lowering the pH to 5.5 (phagolysosome condition), increase or decrease the secretion depending on the protein (Silverman et al., 2010). In present study, MVK release was found to be increased by ~4.2-fold on heat stress (37°C) after 1 h stress treatment in comparison to secretion at 25°C. The temperature induced increase in MVK level must have also been contributed by the global increase in protein level. But, the fold increase value of 4.2 is high and suggests that all of this increment must not have been a part of global increase. Moreover, though acidic pH does not change the bulk of protein release, it led to 6.5-fold change in MVK expression. In extreme acidophiles, an adapted mevalonate pathway is followed, signifying MVK’s involvement in sustaining life in extremely acidic environments (Vinokur et al., 2016) which could be the case in *Leishmania* as well.

A protein can have one function within the cell and another outside it (Jeffery, 1999). A growing number of secretory proteins have been shown to be involved in virulence (**Table 2**). MVK’s partial localization outside glycosome and glycosylated nature could assist in performing an extra function beyond ergosterol synthesis. Besides, it’s different oligomeric state in *L. major* could be used to switch between functions. Thus, to study if MVK performs additional roles apart from ergosterol synthesis in parasite, its role in disease pathogenesis was studied.

**Table 2:** Some *Leishmania* proteins with dual function.

Protein	Organism	Function 1	Function 2	References
Mevalonate Kinase	*L. donovani*	Ergosterol synthesis	Parasite internalization, host immunomodulation	This study
DNA polymerase θ	*L. infantum*	DNA replication	Prevents parasite oxidative damage	Fernández Orgiler et al., 2016
Hexokinase	*L. donovani*	glycolysis	Hemoglobin receptor	Krishnamurthy et al., 2005
Fructose-1,6-bisphosphate aldolase	*L. donovani*	glycolysis	Activation of macrophage SHP-1, macrophage dysfunction	Nandan et al., 2007
Elongation factor-1⍺	*L. donovani*	Protein translation	Activates host SHP-1, inhibits macrophage activity	Nandan et al., 2002
Enolase	*L. mexicana*	Glycolysis and gluconeogenesis	Plasminogen binding protein	Vanegas et al., 2007
Serine protease	*L. donovani*	Peptide maturation and processing	Downregulation of MMP-9 activity	Choudhury et al., 2010b
Protease GP63	*L. major*	Proteolysis duringinfection	Macrophage p38 inactivation, *Leishmania* infectivity	Hallé et al., 2009
HSP100	*L. donovani*	disaggregation of protein aggregates	Modulates exosomal cargo	Silverman et al., 2010
HSP90	*L. donovani*	Maintains proteins native state	Stage differentiation triggering factor	Rolania, 2012
Peroxiredoxin	*L. infantum*	ROS detoxification	Chaperone reservoir, *Leishmania* infectivity	Teixeira et al., 2015
Protein disulfide isomerase	*L. major*	Maintaining reducing intracellular milieu	Intracellular survival of the parasites	Achour et al., 2002

Cytokine response can give a broad prospect of the complicated interaction between host and parasite. Lipopolysaccharide (LPS) induction leads to reduced T_H_1 response (Lauw et al., 2000). Its exposure increases TNFα and interleukin-10 production significantly; and the release of interferon-γ and interleukin-12 remains unaffected (Jansky et al., 2003). Soluble *Leishmania* antigen (SLA) treatment on the other hand, is considered as good control for *Leishmania* specific T_H_1 response (Hailu et al., 2005) and triggers interferon-γ and TNF-α producing cells. Similar results were observed in our study when PBMC were treated with LPS and SLA. Treatment with MVK triggered interleukin-10 and interleukin-4 secretion which is indicative of disease susceptibility (Bhattacharya et al., 2001). Interleukin-10 plays an important role in pathogenesis by inhibiting T_H_-1 mediated response, activation of macrophages, and antigen presentation (De Medeiros et al., 1998; Nylen and Sacks, 2007). Increase in the level of interleukin-4 trigger macrophage in a different manner; and assist in polyamine synthesis, assisting in growth and survival of parasites (Bhattacharya and Ali, 2013). Unexpectedly, there was an increase in the level of interferon-γ release also, though insignificant. It has been shown by Murphy et al. that abundance of interleukin-10 is more important than the level of interferon−γ (Murphy et al., 2001); and interleukin-10 resists interferon-γ induced macrophage activation. Reduced IL-12 secretion in response to MVK treatment would delay T_H_-1 cells development allowing parasite to transform to withstand adverse macrophage conditions. The T_H_1/T_H_2 balance is a determining factor of the consequence of leishmaniasis. Anti-inflammatory to pro-inflammatory cytokines ratios (IL10/IL-12 ratio; IL4/IL-12 and IL-4/IL2 ratio) were found to be significantly higher in LdMVK-treated macrophage compared to untreated and SLA-treated cells. Such high ratios during *Leishmania* infection, specify Th2 dominance, host immunosuppression, and disease progression. Our results are concurrent with the previous studies where mice treated with *L. major* excretory secretory proteins released more IL-4 and IL-10 thereby enhancing parasite survival and disease progression (Tonui et al., 2004). Hence, LdMVK can be among the proteins responsible for transient immunosuppression, favoring parasite internalization inside the host cells.

Earlier observations indicates that proteins involved in parasite host interactions usually bind to the host cell surface (Mortara, 1991). r-MVK was shown to bind to the surface of macrophages in dose-dependent manner by ELISA method. Western blot experiments showing similar results led us to investigate MVK’s role in parasite entry as a protein that binds to host membrane may regulate parasite entry also. Incubation with r-LdMVK and *L. donovani* promastigotes showed ~1.8-fold increase in the number of internalized parasites. Interestingly, the time of addition of rLdMVK plays an important role here. If MVK was pre-incubated with the macrophages 30 min prior, only marginal increase in the internalized parasites was observed (result not shown); but simultaneous treatment with rMVK and parasite had an evident effect. Possible explanation is that MVK causes events to occur during specific time which favors phagocytosis by macrophage. Increase in internalized parasites was also noticed in PBMC-derived macrophages infected with *L. donovani* parasites overexpressing MVK in comparison to macrophages treated with vector control parasites. This clearly confirmed the involvement of MVK in parasite internalization. However, its role in replication or survival of amastigotes inside phagolysosome was not studied. Similar result was observed in the case of *T. cruzi* mevalonate kinase. It was found that MVK positively regulates invasion in *T. cruzi* amastigotes and negatively regulates metacyclic trypomastigotes invasion rate. Entry of *Leishmania* into macrophages is by phagocytosis and is thought to be largely mediated by macrophage. On the contrary, *T. cruzi* infects either by phagocytosis (amastigote) or through lysosomal exocytosis dependent parasite invasion (metacyclic trypomastigotes). The similarity in the entry mechanism of *Leishmania* and *T. cruzi* amastigote, both involving actin cytoskeleton (Mortara, 1991), reinforces the strong similarity between trypanosomatids amastigotes and *Leishmania* in host entry mechanism.

To survive inside macrophages, parasite modulates host cell signaling pathways and its antagonistic and synergistic molecular actions decides the fate of parasite. *Leishmania* causes reciprocal regulation of ERK-1/2 and p38 (Mathur et al., 2004). ERK-1/2 pathway activation causes increased IL-10 production, induces Th-2 type immune response, and parasite survives (Bhardwaj et al., 2010). p38 MAPK activation leads to IL-12 production, guides Th-cell differentiation into Th1-type cell in the favor of host (Dong et al., 2002; Arthur and Ley, 2013). Since, protein phosphorylation is the major regulatory mechanism (Ptacek et al., 2005), phosphorylation kinetics of ERK-1/2 in rLdMVK exposed macrophages was measured and was found to be upregulated transiently post-MVK incubation. Similar results were seen in LPG (Balaraman et al., 2005) and *L. donovani* (Soares-Silva et al., 2016) incubated macrophages. The MVK-induced change in ERK1&2/p38 expression reflects in the high IL-10/IL-12 ratio post MVK treatment, skewing the T cell response towards Th-2 type and in the favor of parasites. ERK also regulates the activation of proteins involved in microfilament remodeling like Cortactin (Navratil et al., 2014) and have also been linked with increased invasion in *T. cruzi* infection (Magdesian et al., 2007). Furthermore, increase in phosphorylation status of cortactin on MVK induction indicates possible involvement of MVK in actin polymerization, since cortactin is the key scaffold for actin regulation (Han et al., 2014). This gives an idea behind the mechanism involved in MVK-mediated parasite internalization and immunosuppression.

We have not shown direct activation of ERK by rLdMVK. MVK mediated indirect phosphorylation of ERK through ERK kinase is also possible. Hence, MVK, apart from phosphorylating the metabolite: mevalonate, might also acts like a protein kinase and phosphorylate ERK-1/2 and cortactin either directly or indirectly. There are several reports of metabolic kinases moonlighting as protein kinases (Lu and Hunter, 2018). Current studies cannot explain how the active site of a metabolic kinase responsible for recognizing a small metabolite and ATP/ADP, can also recognize Ser, Thr, or Tyr residues of protein and phosphorylate it and it needs further investigation. A co-crystal structure of mevalonate kinase bound to its protein substrate could define how an amino acid in the protein substrate can fit in the active site.

**Figure 7:**
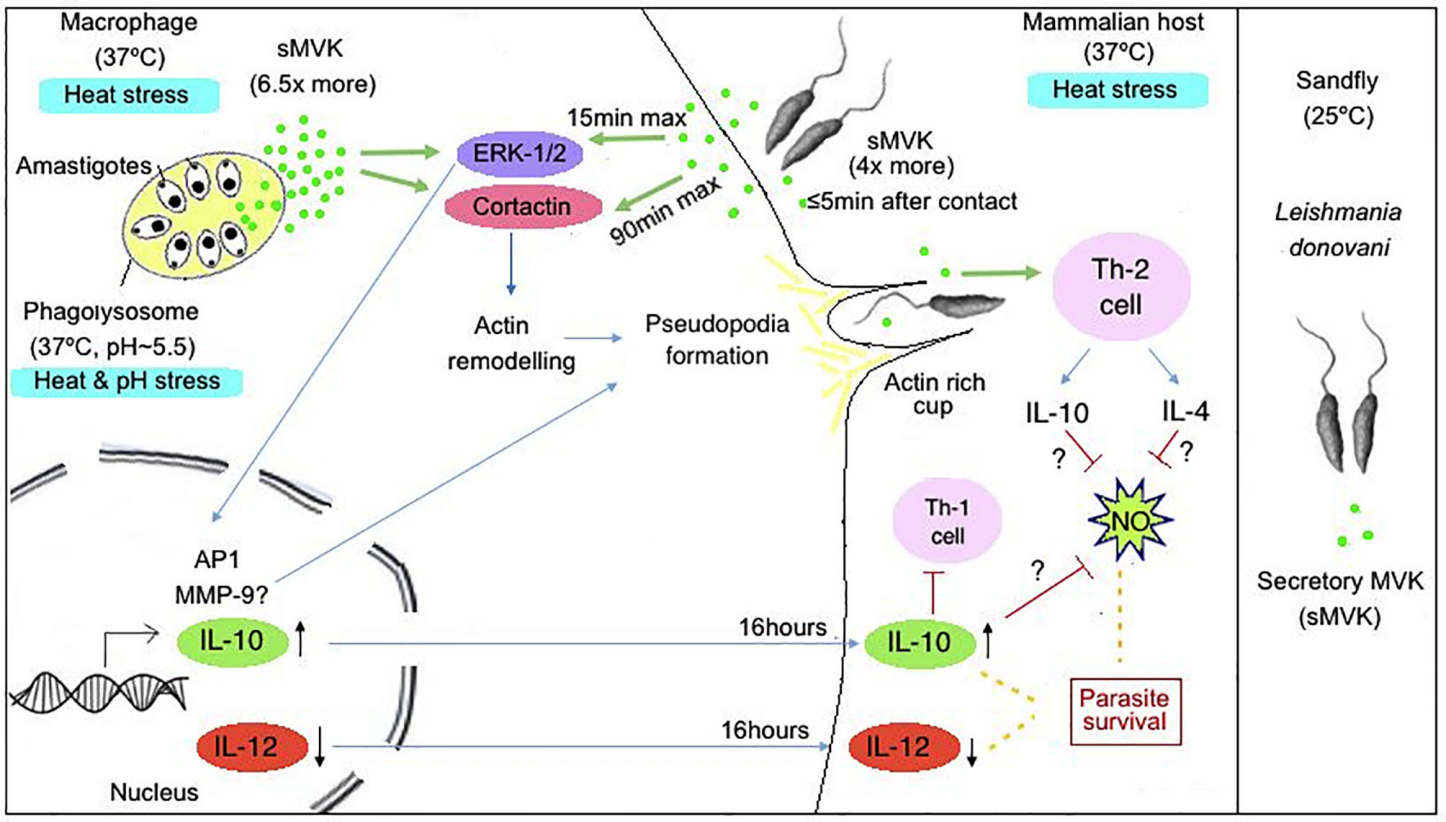
Hypothetical model representing role of LdMVK in infection of macrophage by *Leishmania*. Exposure of *Leishmania* promastigotes to the human host (37°C) leads to increase secretion of MVK (four times more in comparison to parasites at 25°C). Further, *Leishmania* entry inside phagolysosome creates heat and pH stress (37°C, pH~5.5) conditions for the parasite, leading to further increase in MVK secretion (6.5 times more in comparison to parasites at 25°C). Secreted MVK (sMVK) activates host ERK-1/2 which leads to MVK-induced IL-10 production/secretion and suppression of IL-12 production/secretion. MVK also triggers release of IL-4 cytokines and show Th-2 dependent immune response causing immune suppression. Additionally, MVK triggers the activation of Cortactin, promoting actin rich cup formation facilitating parasite entry inside macrophages.

Extracellular secretion of proteins is one of the mechanisms of virulence in *Leishmania*. MVK has to cross two membranes through their journey into phagolysosome. Its secretion from *Leishmania* glycosome occur through non-classical mode of secretion in the form of microvesicles or whole glycosome. It then binds to the membrane of macrophage and is internalized before the internalization of parasite. Once in cytosol, MVK mediates the phosphorylation of host cell signaling pathway proteins. This could lead to cortical actin polymerization, formation of actin rich structure: pseudopodia that would internalize parasite through phagocytosis. MVK-mediated activation of host cell proteins increases and decreases the secretion of interleukin-10 and interleukin-12 respectively. This could help in evasion of the immune system by parasite, facilitating their persistence in host (**Figure 7**).

Together, in this study, we have identified and characterized a novel *Leishmania* secretory protein, MVK, that is involved in internalization of parasite and immunosuppression. It can be predicted that once *Leishmania* sense the changes in environment, the release of MVK is increased that facilitates parasite entry and induce immune suppression through MVK-mediated phosphorylation of host cell components. Overall, these results will improve our understanding on the capabilities of secretory proteins in internalization of parasite and host immunomodulation which may be further explored for their potential importance in diagnosis and immunoprophylaxis.

## Data Availability Statement

The original contributions presented in the study are included in the article/**Supplementary Material**. Further inquiries can be directed to the corresponding author.

## Ethics Statement

The studies involving human participants were reviewed and approved by Institutional Human Ethics Committee (IHEC). The patients/participants provided their written informed consent to participate in this study. The animal study was reviewed and approved by Institutional Animal Ethical Committee, affiliated by The Committee for the Purpose of Control and Supervision of Experiments on Animals (CPCSEA).

## Author Contributions

Conceptualization: TB and PD. Methodology: TB and AS. Validation: MK and AjK. Formal analysis: TB, MD, and KA. Investigation: TB, TS, and AsK. Writing—original draft preparation: TB. Writing—review and editing: TB, SD, AS, and PD. Supervision: PD. All authors contributed to the article and approved the submitted version.

## Funding

TB is a senior research fellow and received funding from Council of Scientific and Industrial Research (CSIR). PD is a J. C. Bose fellow. Indian Council of Medical Research (ICMR) provided infrastructure and facilities.

## Conflict of Interest

The authors declare that the research was conducted in the absence of any commercial or financial relationships that could be construed as a potential conflict of interest.

## Supplementary Material

Supplementary Figure 1 Various parameters for structure validation. **(A)** Structure of LdMVK depicting α-helix, β-sheet, and turn regions illustrated in distinct colors, and representing three predicted N-linked glycosylation sites (N130, N137, N222), and one O-linked glycosylation site (S228). **(B)** Stereochemical properties of the homology model were investigated in Ramachandran plot using PROCHECK analysis. **(C)** ProSA was employed to assess the 3D structure model of protein for errors. Graph represents score plot acquired through ProSA web server. **(D)** The refined model was confirmed by Verify-3D profile analysis method.

## References

[B1] Achour Y. B. Chenik M. Louzir H. Dellagi K. (2002). Identification of a Disulfide Isomerase Protein of Leishmania Major as a Putative Virulence Factor. Infect. Immun. 70 (7), 3576–3585. 10.1128/IAI.70.7.3576-3585.2002 12065498 PMC128112

[B2] Arthur J. S. C. Ley S. C. (2013). Mitogen-Activated Protein Kinases in Innate Immunity. Nat. Rev. Immunol. 13 (9), 679–692. 10.1038/nri3495 23954936

[B3] Balaraman S. Singh V. K. Tewary P. Madhubala R. (2005). Leishmania Lipophosphoglycan Activates the Transcription Factor Activating Protein 1 in J774A.1 Macrophage Through the Extracellular Signal-Related Kinase (ERK) and p38 Mitogen-Activated Protein Kinase. Mol. Biochem. Parasitol. 139 (1), 117–127. 10.1016/j.molbiopara.2004.10.006 15610826

[B4] Bendtsen J. D. Jensen L. J. Blom N. Heijne G. V. Brunak S. (2004). Feature Based Prediction of non-Classical and Leaderless Protein Secretion. Protein Eng. Des. Sel. 17 (4), 349–356. 10.1093/protein/gzh037 15115854

[B5] Bhardwaj S. Srivastava N. Sudan R. Saha B. (2010). Leishmania Interferes With Host Cell Signaling to Devise a Survival Strategy. J. BioMed. Biotechnol. 2010, 109189. 10.1155/2010/109189 20396387 PMC2852600

[B6] Bhattacharya P. Ali N. (2013). Involvement and Interactions of Different Immune Cells and Their Cytokines in Human Visceral Leishmaniasis. Rev. da Sociedade Bras. Med. Trop. 46 (2), 128–134. 10.1590/0037-8682-0022-2012 23559342

[B7] Bhattacharyya S. Ghosh S. Jhonson P. L. Bhattacharya S. K. Majumdar S. (2001). Immunomodulatory Role of interleukin-10 in Visceral Leishmaniasis: Defective Activation of Protein Kinase C-mediated Signal Transduction Events. Infect. Immun. 69 (3), 1499–1507. 10.1128/IAI.69.3.1499-1507.2001 11179319 PMC98048

[B8] Bringaud F. Baltz D. Baltz T. (1998). Functional and Molecular Characterization of a Glycosomal PPi-dependent Enzyme in Trypanosomatids: Pyruvate, Phosphate Dikinase. Proc. Natl. Acad. Sci. 95, 7963–7968. 10.1073/pnas.95.14.7963 9653123 PMC20912

[B9] Brydges S. D. Sherman G. D. Nockemann S. Loyens A. Daubener W. Dubremetz J. F.. (2000). Molecular Characterization of TgMIC5, a Proteolytically Processed Antigen Secreted From the Micronemes of Toxoplasma Gondii. Mol. Biochem. Parasitol. 111, 51–66. 10.1016/S0166-6851(00)00296-6 11087916

[B10] Carrero-Lerida J. Perez-Moreno G. Castillo-Acosta V. M. Ruiz-Perez L. M. Gonzalez-Pacanowska D. (2009). Intracellular Location of the Early Steps of the Isoprenoid Biosynthetic Pathway in the Trypanosomatids Leishmania Major and Trypanosoma Brucei. Int. J. Parasitol. 39, 307–314. 10.1016/j.ijpara.2008.08.012 18848949

[B11] Carruthers V. B. Sherman G. D. Sibley L. D. (2000). The Toxoplasma Adhesive Protein MIC2 is Proteolytically Processed At Multiple Sites by Two Parasite-Derived Proteases. J. Biol. Chem. 275, 14346–14353. 10.1074/jbc.275.19.14346 10799515

[B12] Chakrabarty R. Mukherjee S. Lu H.-G. McGwire B. Chang K.-P. Basu M. (1996). Kinetics of Entry of Virulent and Avirulent Strains of Leishmania Donovani Into Macrophages: A Possible Role of Virulence Molecules (gp63 and LPG). J. Parasitol. 82 (4), 632–635. 10.2307/3283790 8691373

[B13] Choudhury R. Das P. Bhaumik S. K. De T. Chakraborti T. (2010a). In Situ Immunolocalization and Stage-Dependent Expression of a Secretory Serine Protease in Leishmania Donovani and its Role as a Vaccine Candidate. Clin. Vaccine Immunol. 17, 660–667. 10.1128/CVI.00358-09 20106998 PMC2849349

[B14] Choudhury R. Das P. De T. Chakraborti T. (2010b). Immunolocalization and Characterization of Two Novel Proteases in Leishmania Donovani: Putative Roles in Host Invasion and Parasite Development. Biochimie 92, 1274–1286. 10.1016/j.biochi.2010.05.015 20595064

[B15] Connell N. D. Medina-Acosta E. McMaster W. R. Bloom B. R. Russell D. G. (1993). Effective Immunization Against Cutaneous Leishmaniasis With Recombinant Bacille Calmette-Guerin Expressing the Leishmania Surface Proteinase Gp63. Proc. Natl. Acad. Sci. 90, 11473–11477. 10.1073/pnas.90.24.11473 8265576 PMC48006

[B16] Das S. Rani M. Pandey K. Sahoo G. C. Rabidas V. N. Singh D.. (2012). Combination of Paromomycin and Miltefosine Promotes TLR4-dependent Induction of antiLeishmanial Immune Response In Vitro. J. Antimicrob. Chemother. 67, 2373–2378. 10.1093/jac/dks220 22761329

[B17] De Medeiros I. M. Castelo A. Salomao R. (1998). Presence of Circulating Levels of Interferon-Gamma, interleukin-10 and Tumor Necrosis Factor-Alpha in Patients With Visceral Leishmaniasis. Rev. Inst. Med. Trop. Sao Paulo 40, 31–34. 10.1590/S0036-46651998000100007 9713135

[B18] Dong C. Davis R. J. Flavell R. A. (2002). MAP Kinases in the Immune Response. Annu. Rev. Immunol. 20 (1), 55–72. 10.1146/annurev.immunol.20.091301.131133 11861597

[B19] Dorsey J. K. Porter J. W. (1968). The Inhibition of Mevalonic Kinase by Geranyl and Farnesyl Pyrophosphates. J. Biol. Chem. 243, 4667–4670. 10.1016/S0021-9258(18)93170-4 4300840

[B20] Douanne N. Dong G. Douanne M. Olivier M. Fernandez-Prada C. (2020). Unravelling the Proteomic Signature of Extracellular Vesicles Released by Drug-Resistant Leishmania Infantum Parasites. PloS Neglected Trop. Dis. 14 (7), p.e0008439. 10.1371/journal.pntd.0008439 PMC7365475 32628683

[B21] Duarte D. P. Ferreira É. R. Lima F. M. Batista F. De Groote M. Horjales E.. (2018). Molecular Characterization of Trypanosoma Evansi Mevalonate Kinase (Temvk). Front. Cell. Infect. Microbiol. 8, 223. 10.3389/fcimb.2018.00223 30042928 PMC6048237

[B22] Escrevente C. Keller S. Altevogt P. Costa J. (2011). Interaction and Uptake of Exosomes by Ovarian Cancer Cells. BMC Cancer 11, 108. 10.1186/1471-2407-11-108 21439085 PMC3072949

[B23] Fernandez-Orgiler A. Martinez-Jimenez M. I. Alonso A. Alcolea P. J. Requena J. M. Thomas M. C.. (2016). A Putative Leishmania DNA Polymerase Theta Protects the Parasite Against Oxidative Damage. Nucleic Acids Res. 44, 4855–4870. 10.1093/nar/gkw346 27131366 PMC4889957

[B24] Ferreira É. R. Horjales E. Bonfim-Melo A. Cortez C. Da Silva C. V. De Groote M.. (2016). Unique Behavior of Trypanosoma Cruzi Mevalonate Kinase: A Conserved Glycosomal Enzyme Involved in Host Cell Invasion and Signaling. Sci. Rep. 6, 1–13. 10.1038/srep24610 27113535 PMC4845012

[B25] Forrest D. M. Batista M. Marchini F. K. Tempone A. J. Traub-Csekö Y. M. (2020). Proteomic Analysis of Exosomes Derived From Procyclic and Metacyclic-Like Cultured Leishmania Infantum Chagasi. J. Proteomics 227, p.103902. 10.1016/j.jprot.2020.103902 32673755

[B26] Fu Z. Wang M. Potter D. Miziorko H. M. Kim J.-J. P. (2002). The Structure of a Binary Complex Between a Mammalian Mevalonate Kinase and ATP Insights Into the Reaction Mechanism and Human Inherited Disease. J. Biol. Chem. 277, 18134–18142. 10.1074/jbc.M200912200 11877411

[B27] Geiger A. Hirtz C. Becue T. Bellard E. Centeno D. Gargani D.. (2010). Exocytosis and Protein Secretion in Trypanosoma. BMC Microbiol. 10, 20. 10.1186/1471-2180-10-20 20102621 PMC3224696

[B28] Gerlach J. Q. Kruger A. Gallogly S. Hanley S. A. Hogan M. C. Ward C. J.. (2013). Surface Glycosylation Profiles of Urine Extracellular Vesicles. PloS One 8, e74801. 10.1371/journal.pone.0074801 24069349 PMC3777961

[B29] Hailu A. van Baarle D. Knol G. J. Berhe N. Miedema F. Kager P. A. (2005). T Cell Subset and Cytokine Profiles in Human Visceral Leishmaniasis During Active and Asymptomatic or Sub-Clinical Infection With Leishmania Donovani. Clin. Immunol. 117 (2), pp.182–pp.191. 10.1016/j.clim.2005.06.015 16125466

[B30] Hallé M. Gomez M. A. Stuible M. Shimizu H. McMaster W. R. Olivier M.. (2009). The Leishmania Surface Protease GP63 Cleaves Multiple Intracellular Proteins and Actively Participates in p38 Mitogen-Activated Protein Kinase Inactivation. J. Biol. Chem. 284 (11), 6893–6908. 10.1074/jbc.M805861200 19064994 PMC2652307

[B31] Han S. P. Gambin Y. Gomez G. A. Verma S. Giles N. Michael M.. (2014). Cortactin scaffolds Arp2/3 and WAVE2 at the epithelial zonula adherens. J. Biol. Chem. 289 (11), 7764–7775. 24469447 10.1074/jbc.M113.544478 PMC3953287

[B32] Hassani K. Antoniak E. Jardim A. Olivier M. (2011). Temperature-Induced Protein Secretion by Leishmania Mexicana Modulates Macrophage Signalling and Function. PloS One 6, e18724. 10.1371/journal.pone.0018724 21559274 PMC3086886

[B33] Henneman L. van Cruchten A. G. Kulik W. Waterham H. R. (2011). Inhibition of the Isoprenoid Biosynthesis Pathway; Detection of Intermediates by UPLC-MS/MS. Biochim. Biophys. Acta 1811, 227–233. 10.1016/j.bbalip.2011.01.002 21237288

[B34] Huynh M. H. Carruthers V. B. (2016). A Toxoplasma Gondii Ortholog of Plasmodium Gama Contributes to Parasite Attachment and Cell Invasion. mSphere 1 (1). 10.1128/mSphere.00012-16 PMC4863602 27303694

[B35] Jaffe C. L. Dwyer D. M. (2003). Extracellular Release of the Surface Metalloprotease, gp63, From Leishmania and Insect Trypanosomatids. Parasitol. Res. 91 (3), 229–237. 10.1007/s00436-003-0960-0 12923634

[B36] Jansky L. Reymanova P. Kopecky J. (2003). Dynamics of Cytokine Production in Human Peripheral Blood Mononuclear Cells Stimulated by LPS, or Infected by Borrelia. Physiol. Res. 52 (5), pp.593–pp.598. 14535835

[B37] Jardim A. Hardie D. B. Boitz J. Borchers C. H. (2018). Proteomic Profiling of Leishmania Donovani Promastigote Subcellular Organelles. J. Proteome Res. 17 (3), 1194–1215. 10.1021/acs.jproteome.7b00817 29332401

[B38] Jeffery C. J. (1999). Moonlighting Proteins. Trends Biochem. Sci. 24, 8–11. 10.1016/S0968-0004(98)01335-8 10087914

[B39] Joshi M. B. Mallinson D. J. Dwyer D. M. (2004). The Human Pathogen Leishmania Donovani Secretes a Histidine Acid Phosphatase Activity That is Resistant to Proteolytic Degradation. J. Eukaryotic Microbiol. 51 (1), pp.108–pp.112. 10.1111/j.1550-7408.2004.tb00171.x 15068272

[B40] Kaul P. Malla N. Kaur S. Mahajan R. C. Ganguly N. K. (2000). Evaluation of a 200-kDa Amastigote-Specific Antigen of L. Donovani by Enzyme-Linked Immunosorbent Assay (ELISA) for the Diagnosis of Visceral Leishmaniasis. Trans. R Soc. Trop. Med. Hyg. 94, 173–175. 10.1016/S0035-9203(00)90264-5 10897360

[B41] Kielian M. C. Steinman R. M. Cohn Z. A. (1982). Intralysosomal Accumulation of Polyanions. I. Fusion of Pinocytic and Phagocytic Vacuoles With Secondary Lysosomes. J. Cell Biol. 93, 866–874. 10.1083/jcb.93.3.866 6181074 PMC2112160

[B42] Kima P. E. Bonilla J. A. Cho E. Ndjamen B. Canton J. Leal N.. (2010). Identification of Leishmania Proteins Preferentially Released in Infected Cells Using Change Mediated Antigen Technology (CMAT). PloS Negl. Trop. Dis. 4 (10), p.e842. 10.1371/journal.pntd.0000842 PMC2950143 20957202

[B43] Kink J. A. Chang K.-P. (1987). Tunicamycin-Resistant Leishmania Mexicana Amazonensis: Expression of Virulence Associated With an Increased Activity of N-acetylglucosaminyltransferase and Amplification of its Presumptive Gene. Proc. Natl. Acad. Sci. 84, 1253–1257. 10.1073/Fpnas.84.5.1253 2950522 PMC304405

[B44] Krishnamurthy G. Vikram R. Singh S. B. Patel N. Agarwal S. Mukhopadhyay G.. (2005). Hemoglobin Receptor in Leishmania is a Hexokinase Located in the Flagellar Pocket. J. Biol. Chem. 280 (7), 5884–5891. 10.1074/jbc.M411845200 15579464

[B45] Kumar A. Das S. Mandal A. Verma S. Abhishek K. Kumar A. (2018). Leishmania infection activates host mTOR for its survival by M2 macrophage polarization. Parasite Immunol. 40 (11), p.e12586. 10.1111/pim.12586 30187512

[B46] Lauw F. N. ten Hove T. Dekkers P. E. de Jonge E. van Deventer S. J. van der Poll T. (2000). Reduced Th1, But Not Th2, Cytokine Production by Lymphocytes After In Vivo Exposure of Healthy Subjects to Endotoxin. Infect. Immun. 68 (3), 1014–1018. 10.1128/IAI.68.3.1014-1018.2000 10678901 PMC97242

[B47] Lu Z. Hunter T. (2018). Metabolic Kinases Moonlighting as Protein Kinases. Trends Biochem. Sci. 43 (4), 301–310. 10.1016/j.tibs.2018.01.006 29463470 PMC5879014

[B48] Magdesian M. H. Tonelli R. R. Fessel M. R. Silveira M. S. Schumacher R. I. Linden R.. (2007). A Conserved Domain of the gp85/trans-sialidase Family Activates Host Cell Extracellular Signal-Regulated Kinase and Facilitates Trypanosoma Cruzi Infection. Exp. Cell Res. 313 (1), 210–218. 10.1016/j.yexcr.2006.10.008 17101128

[B49] Mathur R. K. Awasthi A. Wadhone P. Ramanamurthy B. Saha B. (2004). Reciprocal CD40 Signals Through p38MAPK and ERK-1/2 Induce Counteracting Immune Responses. Nat. Med. 10 (5), 540–544. 10.1038/nm1045 15107845

[B50] McConville M. J. Collidge T. A. Ferguson M. A. Schneider P. (1993). The Glycoinositol Phospholipids of Leishmania Mexicana Promastigotes. Evidence for the Presence of Three Distinct Pathways of Glycolipid Biosynthesis. J. Biol. Chem. 268, 15595–15604. 10.1016/S0021-9258(18)82298-0 8340385

[B51] Miettinen T. P. Björklund M. (2016). The Mevalonate Pathway as a Metabolic Requirement for Autophagy–Implications for Growth Control, Proteostasis, and Disease. Mol. Cell. Oncol. 3, e1143546. 10.1080/23723556.2016.1143546 27314093 PMC4909433

[B52] Mortara R. A. (1991). Trypanosoma Cruzi: Amastigotes and Trypomastigotes Interact With Different Structures on the Surface of HeLa Cells. Exp. Parasitol. 73, 1–14. 10.1016/0014-4894(91)90002-E 2055296

[B53] Murphy M. L. Wille U. Villegas E. N. Hunter C. A. Farrell J. P. (2001). Il-10 Mediates Susceptibility to *Leishmania Donovani* Infection. Eur. J. Immunol. 31 (10), pp.2848–2856. 10.1002/1521-4141(2001010)31:10<2848::AID-IMMU2848>3.0.CO;2-T 11592059

[B54] Nandan D. Tran T. Trinh E. Silverman J. M. Lopez M. (2007). Identification of Leishmania fructose-1, 6-Bisphosphate Aldolase as a Novel Activator of Host Macrophage Src Homology 2 Domain Containing Protein Tyrosine Phosphatase SHP-1. Biochem. Biophys. Res. Commun. 364 (3), 601–607. 10.1016/j.bbrc.2007.10.065 18028878

[B55] Nandan D. Yi T. Lopez M. Lai C. Reiner N. E. (2002). Leishmania EF-1α Activates the Src Homology 2 Domain Containing Tyrosine Phosphatase SHP-1 Leading to Macrophage Deactivation. J. Biol. Chem. 277, 50190–50197. 10.1074/jbc.M209210200 12384497

[B56] Navratil A. M. Dozier M. G. Whitesell J. D. Clay C. M. Roberson M. S. (2014). Role of Cortactin in Dynamic Actin Remodeling Events in Gonadotrope Cells. Endocrinology 155 (2), 548–557. 10.1074/jbc.m209210200 24274984 PMC3891938

[B57] Nolan T. J. Farrell J. P. (1985). Inhibition of In Vivo and In Vitro Infectivity ofLeishmania Donovani by Tunicamycin. Mol. Biochem. Parasitol. 16, 127–135. 10.1016/0166-6851(85)90081-7 4033693

[B58] Nothaft H. Szymanski C. M. (2010). Protein Glycosylation in Bacteria: Sweeter Than Ever. Nat. Rev. Microbiol. 8 (11), pp.765–pp.778. 10.1038/nrmicro2383 20948550

[B59] Nylen S. Sacks D. (2007). Interleukin-10 and the Pathogenesis of Human Visceral Leishmaniasis. Trends Immunol. 28, 378–384. 10.1016/j.it.2007.07.004 17689290

[B60] Ptacek J. Devgan G. Michaud G. Zhu H. Zhu X. Fasolo J.. (2005). Global Analysis of Protein Phosphorylation in Yeast. Nature 438 (7068), 679–684. 10.1038/nature04187 16319894

[B61] Rolanía J. M. R. (2012). “The Stressful Life of Pathogenic Leishmania Species,” in Stress Response in Microbiology (United Kingdom: Caister Academic Press), 323–346.

[B62] Sgraja T. Smith T. K. Hunter W. N. (2007). Structure, Substrate Recognition and Reactivity of Leishmania Major Mevalonate Kinase. BMC Struct. Biol. 7, 20. 10.1186/1472-6807-7-20 17397541 PMC1851959

[B63] Silverman J. M. Chan S. K. Robinson D. P. Dwyer D. M. Nandan D. Foster L. J.. (2008). Proteomic Analysis of the Secretome of Leishmania Donovani. Genome Biol. 9, R35. 10.1186/gb-2008-9-2-r35 18282296 PMC2374696

[B64] Silverman J. M. Clos J. de’Oliveira C. C. Shirvani O. Fang Y. Wang C.. (2010). An Exosome-Based Secretion Pathway is Responsible for Protein Export From Leishmania and Communication With Macrophages. J. Cell Sci. 123, 842–852. 10.1242/jcs.056465 20159964

[B65] Soares-Silva M. Diniz F. F. Gomes G. N. Bahia D. (2016). The Mitogen-Activated Protein Kinase (MAPK) Pathway: Role in Immune Evasion by Trypanosomatids. Front. Microbiol. 7, 183. 10.3389/fmicb.2016.00183 26941717 PMC4764696

[B66] Staubach S. Schadewaldt P. Wendel U. Nohroudi K. Hanisch F. G. (2012). Differential Glycomics of Epithelial Membrane Glycoproteins From Urinary Exovesicles Reveals Shifts Toward Complex-Type N-glycosylation in Classical Galactosemia. J. Proteome Res. 11, 906–916. 10.1021/pr200711w 22087537

[B67] Teixeira F. Castro H. Cruz T. Tse E. Koldewey P. Southworth D. R.. (2015). Mitochondrial Peroxiredoxin Functions as Crucial Chaperone Reservoir in Leishmania Infantum. Proc. Natl. Acad. Sci. 112 (7), E616–E624. 10.1242/jcs.056465 25646478 PMC4343147

[B68] Tonui W. K. Mejia J. S. Hochberg L. Mbow M. L. Ryan J. R. Chan A. S.. (2004). Immunization With Leishmania Major Exogenous Antigens Protects Susceptible BALB/c Mice Against Challenge Infection With L. Major. Infect. Immun. 72, 5654–5661. 10.1128/iai.72.10.5654-5661.2004 15385463 PMC517560

[B69] Vanegas G. Quiñones W. Carrasco-López C. Concepción J. L. Albericio F. Avilán L. (2007). Enolase as a Plasminogen Binding Protein in Leishmania Mexicana. Parasitol. Res. 101, 1511–1516. 10.1007/s00436-007-0668-7 17653767

[B70] Vinokur J. M. Cummins M. C. Korman T. P. Bowie J. U. (2016). An Adaptation to Life in Acid Through a Novel Mevalonate Pathway. Sci. Rep. 6, 1–11. 10.1038/srep39737 28004831 PMC5177888

[B71] Wang C. Eufemi M. Turano C. Giartosio A. (1996). Influence of the Carbohydrate Moiety on the Stability of Glycoproteins. Biochemistry 35, 7299–7307. 10.1021/bi9517704 8652506

[B72] Winter G. Fuchs M. McConville M. J. Stierhof Y. D. Overath P. (1994). Surface Antigens of Leishmania Mexicana Amastigotes: Characterization of Glycoinositol Phospholipids and a Macrophage-Derived Glycosphingolipid. J. Cell Sci. 107 (Pt 9), 2471–2482. 10.1242/jcs.107.9.2471 7844164

[B73] Zylbersztejn A. M. B. de Morais C. G. V. Lima A. K. C. Souza J. E. d O. Lopes A. H.. (2015). Ck2 Secreted by Leishmania Braziliensis Mediates Macrophage Association Invasion: A Comparative Study Between Virulent and Avirulent Promastigotes. BioMed. Res. Int. 2015, 167323–167323. 10.1155/2015/167323 PMC4450227 26120579

