# Supplementary figures and images for: Leishmania donovani Secretory Mevalonate Kinase Regulates Host Immune Response and Facilitates Phagocytosis
Source: Front Cell Infect Microbiol. 2021 Apr 26;11:641985. doi: 10.3389/fcimb.2021.641985 (PMC8110032; doi:10.3389/fcimb.2021.641985)

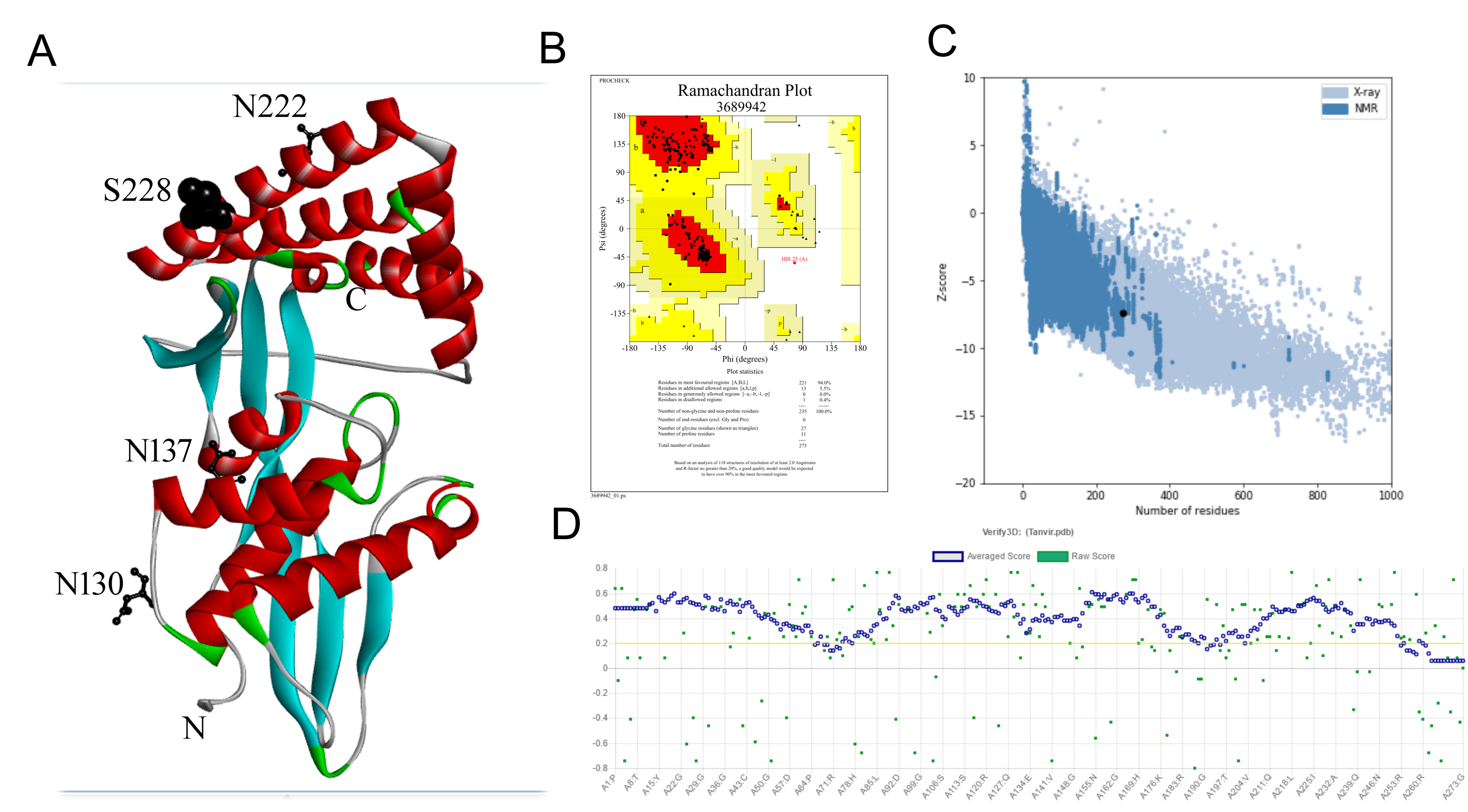

Supplement: Supplementary Figure 1 — Various parameters for structure validation. (A) Structure of LdMVK depicting α-helix, β-sheet, and turn regions illustrated in distinct colors, and representing three predicted N-linked glycosylation sites (N130, N137, N222), and one O-linked glycosylation site (S228). (B) Stereochemical properties of the homology model were investigated in Ramachandran plot using PROCHECK analysis. (C) ProSA was employed to assess the 3D structure model of protein for errors. Graph represents score plot acquired through ProSA web server. (D) The refined model was confirmed by Verify-3D profile analysis method. [file Image_1.tiff]
